# Thirty loci identified for heart rate response to exercise and recovery implicate autonomic nervous system

**DOI:** 10.1038/s41467-018-04148-1

**Published:** 2018-05-16

**Authors:** Julia Ramírez, Stefan van Duijvenboden, Ioanna Ntalla, Borbala Mifsud, Helen R Warren, Evan Tzanis, Michele Orini, Andrew Tinker, Pier D. Lambiase, Patricia B. Munroe

**Affiliations:** 1 Clinical Pharmacology, William Harvey Research Institute, Barts and The London School of Medicine and Dentistry, Queen Mary University of London, London, EC1M 6BQ UK; 2 Institute of Cardiovascular Science, University College London, London, WC1E 6BT UK; 3 NIHR Barts Cardiovascular Biomedical Research Centre, Barts and The London School of Medicine and Dentistry, Queen Mary University of London, London, EC1M 6BQ UK; 4 Barts Heart Centre, St Bartholomews Hospital, London, EC1A 7BE UK; 5 Mechanical Engineering Department, University College London, London, WC1E 6BT UK

## Abstract

Impaired capacity to increase heart rate (HR) during exercise (ΔHR^ex^), and a reduced rate of recovery post-exercise (ΔHR^rec^) are associated with higher cardiovascular mortality rates. Currently, the genetic basis of both phenotypes remains to be elucidated. We conduct genome-wide association studies (GWASs) for ΔHR^ex^ and ΔHR^rec^ in ~40,000 individuals, followed by replication in ~27,000 independent samples, all from UK Biobank. Six and seven single-nucleotide polymorphisms for ΔHR^ex^ and ΔHR^rec^, respectively, formally replicate. In a full data set GWAS, eight further loci for ΔHR^ex^ and nine for ΔHR^rec^ are genome-wide significant (*P* ≤ 5 × 10^−8^). In total, 30 loci are discovered, 8 being common across traits. Processes of neural development and modulation of adrenergic activity by the autonomic nervous system are enriched in these results. Our findings reinforce current understanding of HR response to exercise and recovery and could guide future studies evaluating its contribution to cardiovascular risk prediction.

## Abstract (web summary)

Genome-wide association studies have identified multiple loci for resting heart rate (HR) but the genetic factors associated with HR increase during and HR recovery after exercise are less well studied. Here, the authors examine both traits in a two-stage GWAS design in up to 67,257 individuals from UK Biobank.

## Introduction

Increased resting heart rate (HR) has been demonstrated to be an independent risk factor for cardiovascular mortality even in healthy individuals^1–3^. The heritability of resting HR is estimated to be 26–32% from family studies^4,5^, and 55–63% in twin studies^6^. Consequently, genetic association studies have been undertaken to detect genetic determinants of resting HR. Seventy-three loci have been identified to date^7–14^, and a recent study including 64 loci that were robustly validated accounted for 2.5% of the trait variance^7^.

Less is known about the genetic basis of both HR response to exercise and to recovery. Impaired capacity to increase HR during exercise (chronotropic incompetence) and a reduced rate of recovery post exercise (sustained sympathetic activation) have been associated with all-cause and cardiovascular mortality in both healthy individuals and those with heart failure^15–18^. Furthermore, the haemodynamic response to exercise and to recovery is suggested to have a significant heritable component^19^. A genome-wide association study (GWAS) including up to 100,000 variants has been undertaken for peak HR and recovery HR during an exercise test in 1238 individuals from the Framingham Heart Study. No genome-wide significant (GWS) loci were found for any of the two traits^20^. The genetic basis of HR response to exercise and to recovery has only recently been studied^21^.

The identification of genetic loci may aid prognosis and inform the development of new HR modulatory agents, which are known to reduce mortality in heart failure^22^. In addition, it could provide new insights into the development of arrhythmias including autonomic effects modulating the conduction-repolarization dynamics of the myocardium with relevance to new anti-arrhythmic drug targets.

The aim of this study is to discover single-nucleotide polymorphisms (SNPs) associated with the responses of HR to exercise and to recovery. We analyse automated HR measurements and electrocardiogram (ECG) recordings from individuals who participated in the ‘Cardio test’, thereafter referred to as the exercise test, from the UK Biobank (UKB) study. Two phenotypes are studied: (1) HR response to exercise (ΔHR^ex^) and (2) HR response to recovery (1 min post-exercise, ΔHR^rec^*)*. We perform a discovery GWAS for each trait in ~40,000 individuals of European ancestry, and a validation experiment in the remaining ~27,000 samples, all within UKB. We next conduct a full data set GWAS including all individuals (~67,000). Bioinformatics analyses of the newly identified loci for each trait provide new insights into potential causal candidate genes and biological mechanisms.

Six and seven SNPs for ΔHR^ex^ and ΔHR^rec^, respectively, formally replicate. In a full data set GWAS, eight further loci for ΔHR^ex^ and nine for ΔHR^rec^ are genome-wide significant (*P* ≤ 5 × 10^−8^). In total, 30 loci are discovered, 8 being common across traits. Processes of neural development and modulation of adrenergic activity by the autonomic nervous system are enriched in these results. Our findings reinforce current understanding of HR response to exercise and recovery and will guide future studies evaluating its contribution to cardiovascular risk prediction.

## Results

### Identification of loci associated with ΔHR^ex^ and ΔHR^rec^

**Fig. 1:**
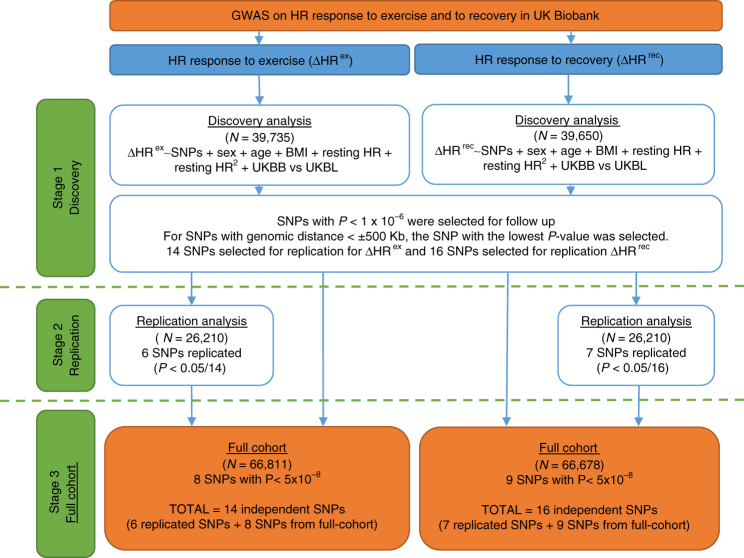
Flowchart of the analytical 3-stage approach. HR heart rate, ΔHR^ex^ changes in HR during exercise, ΔHR^rec^ changes in HR 1 min post-exercise, SNP: single-nucleotide polymorphism, BMI: body mass index, UKBB: UK Biobank genetic array, UKBL: UK BiLEVE genetic array, UKBB vs. UKBL: binary indicator variable for UK Biobank versus UK BiLEVE to adjust for the different genotyping chips, MAF: minor allele frequency, HWE: Hardy–Weinberg Equilibrium, INFO: parameter indicating the quality of imputation, HRC: Haplotype Reference Consortium

An overview of the study design is provided in Fig. 1. The demographics of the discovery and replication samples did not significantly differ (Supplementary Table 1). In a discovery phase, genome-wide association results of ~7.8 million SNPs from ~40,000 individuals of European ancestry from UKB were analysed for each trait, ΔHR^ex^ and ΔHR^rec^ (Methods; Supplementary Table 2 and Supplementary Data 1). Three genome-wide significant (*P* ≤ 5 × 10^−8^) loci were found for ΔHR^ex^, and six for ΔHR^rec^ (Supplementary Tables 2 and Supplementary Data 1).

Using a significance threshold of *P* < 1 × 10^−6^, 14 and 16 variants (considering one lead SNP per 1 Mb region) were taken forward for replication for ΔHR^ex^ and ΔHR^rec^, respectively, in ~27,000 independent samples from UKB (Methods).

Six of the selected SNPs for ΔHR^ex^ formally replicated (*P* ≤ 0.05/14 = 0.00356) and all had concordant directions of effect (Table 1). Regarding ΔHR^rec^, 7 SNPs were formally replicated (*P* ≤ 0.05/16 = 0.0031), all showing as well concordant directions of effect (Table 2). The range of magnitude of the β estimates for these SNPs was 0.29–0.86 bpm per allele for ΔHR^ex^ (Table 1) and 0.26–0.67 bpm for ΔHR^rec^ (Table 2).

**Table 1:** Loci associated with HR response to exercise

						**Discovery**				**Replication**				**Full**			
**Locus**	**SNP**	**CHR**	**BP**	**EA**	**EAF**	**P**	**N**	**β**	**SE**	**P**	**N**	**β**	**SE**	**P**	**N**	**β**	**SE**
RNF220*	rs272564	1	45012273	A	0.716	4.30E-05	38757	0.360	0.088	1.10E-08	25614	0.619	0.108	7.40E-12	65166	0.462	0.068
**CCDC141**	**rs10497529**	**2**	**179839888**	**G**	**0.964**	**7.80E-07**	**39735**	**−1.049**	**0.212**	**9.10E-04**	**26261**	**−0.860**	**0.259**	**2.50E-09**	**66811**	**−0.968**	**0.162**
SCN10A*	rs7433723	3	38784957	G	0.420	4.80E-06	39674	**−**0.364	0.080	7.40E-04	26221	**−**0.333	0.099	4.50E-08	66709	**−**0.335	0.061
SNCA1P*	**rs4836027**	**5**	**121866990**	**T**	**0.693**	**5.70E-13**	**39214**	**0.617**	**0.086**	**1.20E-08**	**25917**	**0.599**	**0.105**	**9.90E-21**	**65935**	**0.613**	**0.066**
PPIL1	rs236352	6	36817113	A	0.341	4.20E-06	39213	0.383	0.083	9.40E-05	25916	0.401	0.103	6.40E-10	65933	0.395	0.064
CAV2*	rs28495552	7	116113744	C	0.485	6.40E-06	39671	**−**0.355	0.079	1.70E-06	26219	**−**0.467	0.097	2.80E-11	66703	**−**0.403	0.061
**RP1L1**	**rs58065122**	**8**	**10526186**	**G**	**0.582**	**6.60E-08**	**39187**	**−0.434**	**0.080**	**2.90E-03**	**25899**	**−0.294**	**0.099**	**3.90E-10**	**65889**	**−0.385**	**0.062**
PAX2*	rs11190709	10	102552663	G	0.112	1.10E-04	39359	0.484	0.125	2.70E-08	26013	0.852	0.153	1.30E-11	66180	0.649	0.096
SOX5*	**rs4246224**	**12**	**24784139**	**G**	**0.851**	**2.30E-10**	**39716**	**−0.702**	**0.111**	**5.40E-06**	**26248**	**−0.615**	**0.135**	**1.80E-14**	**66778**	**−0.649**	**0.085**
SYT10*	**rs1343676**	**12**	**33537387**	**T**	**0.494**	**2.20E-07**	**39537**	**−0.408**	**0.079**	**3.10E-05**	**26130**	**−0.404**	**0.097**	**1.50E-11**	**66477**	**−0.407**	**0.060**
HMGA2	rs1480470	12	66412130	A	0.371	7.90E-06	39139	0.367	0.082	3.30E-03	25867	0.294	0.100	3.40E-08	65810	0.347	0.063
MCTP2*	**rs12906962**	**15**	**95312071**	**T**	**0.680**	**5.60E-09**	**39222**	**0.496**	**0.085**	**1.10E-05**	**25922**	**0.461**	**0.105**	**3.50E-13**	**65949**	**0.475**	**0.065**
TCF4	rs1125313	18	52859261	A	0.501	1.10E-05	39348	**−**0.349	0.079	1.30E-04	26005	**−**0.375	0.098	3.90E-09	66161	**−**0.359	0.061
POP4	rs7255293	19	30104198	A	0.580	9.60E-06	39405	0.354	0.080	3.40E-04	26043	0.352	0.098	3.20E-09	66257	0.363	0.061

The locus name indicates the gene that is in the closest proximity to the most associated SNP

Replicated SNPs are indicated in bold type

* indicates the SNP is the same or in high LD (*r*^2^ > 0.8) with a SNP associated with the other HR response trait

*SNP* single-nucleotide polymorphism, *CHR* Chromosome, *BP* Base pair Position, based on HG build 19, *EA* Effect allele, *EAF* Effect allele frequency from discovery data set, *β* Beta in beats per minute, *SE* Standard Error, *N* effective number of participants, *P* P-value

**Table 2:** Loci associated with HR response to recovery

						**Discovery**				**Replication**				**Full**			
**Locus**	**SNP**	**CHR**	**BP**	**EA**	**EAF**	**P**	**N**	**β**	**SE**	**P**	**N**	**β**	**SE**	**P**	**N**	**β**	**SE**
RNF220*	rs272564	1	45012273	A	0.716	5.20E-06	38674	0.359	0.079	5.00E-05	25571	0.391	0.096	8.80E-10	65036	0.370	0.060
BCL11A^a^	rs1372876	2	60025963	A	0.414	1.10E-06	38731	−0.350	0.072	2.E-03	25608	−0.271	0.088	3.30E-09	65133	−0.326	0.055
SCN10A*	rs6795970	3	38766675	A	0.404	4.80E-07	39650	−0.360	0.072	6.E-03	26216	−0.244	0.088	2.60E-08	66678	−0.306	0.055
**CNTN3**	**rs6549649**	**3**	**74786491**	**G**	**0.559**	**1.20E-07**	**39438**	−**0.375**	**0.071**	**1.50E-03**	**26076**	−**0.275**	**0.087**	**1.40E-09**	**66321**	−**0.328**	**0.054**
SNCA1P*	rs1993875	5	121869310	G	0.697	1.00E-05	39185	0.339	0.077	1.80E-04	25908	0.352	0.094	9.50E-09	65895	0.338	0.059
ACHE	rs3757868	7	100482720	G	0.815	1.10E-05	39602	0.396	0.090	1.50E-06	26184	0.539	0.112	6.90E-11	66597	0.454	0.070
CAV2*	**rs2109514**	**7**	**116159961**	**G**	**0.501**	**3.10E-08**	**39462**	−**0.391**	**0.071**	**2.80E-03**	**26092**	−**0.259**	**0.087**	**7.10E-10**	**66362**	−**0.334**	**0.054**
CHRM2	rs6943656	7	136639436	A	0.842	1.40E-06	39113	0.466	0.097	1.00E-04	25861	0.462	0.119	2.30E-10	65775	0.470	0.074
PAX2*	**rs4917911**	**10**	**102559421**	**G**	**0.111**	**2.60E-08**	**39470**	**0.622**	**0.112**	**7.10E-07**	**26097**	**0.673**	**0.136**	**6.60E-15**	**66376**	**0.665**	**0.085**
SOX5*	**rs112630705**	**12**	**24773919**	**G**	**0.851**	**1.10E-08**	**39625**	−**0.565**	**0.099**	**9.70E-04**	**26199**	−**0.397**	**0.120**	**3.20E-11**	**66636**	−**0.502**	**0.076**
SYT10*	**rs2218650**	**12**	**33734783**	**A**	**0.641**	**2.50E-18**	**39522**	**0.642**	**0.073**	**1.40E-09**	**26131**	**0.545**	**0.090**	**1.10E-26**	**66463**	**0.602**	**0.056**
**ALG10B**	**rs4533105**	**12**	**38214611**	**T**	**0.570**	**1.00E-08**	**38916**	**0.411**	**0.072**	**8.20E-06**	**25731**	**0.392**	**0.088**	**1.90E-13**	**65444**	**0.404**	**0.055**
**MED13L**	**rs11067773**	**12**	**116228495**	**T**	**0.910**	**4.70E-07**	**39403**	**0.621**	**0.123**	**2.80E-05**	**26053**	**0.635**	**0.152**	**3.10E-11**	**66262**	**0.628**	**0.095**
RGS6b	rs150330648	14	72844765	G	0.987	1.70E-07	35768	1.734	0.331	6.10E-02	23649	0.765	0.408	4.30E-08	60150	1.395	0.255
MCTP2*	rs12906962	15	95312071	T	0.680	2.40E-08	39139	0.425	0.076	2.E-02	25878	0.218	0.093	5.10E-09	65818	0.341	0.058
NDUFA11	rs12974991	19	5894584	G	0.912	3.30E-05	39532	0.512	0.123	5.20E-05	26138	0.616	0.152	2.10E-09	66479	0.568	0.095

The locus name indicates the gene that is in the closest proximity to the most associated SNP

Replicated SNPs are indicated in bold type

* indicates the SNP is the same or in high LD (*r*^2^ > 0.8) with a SNP associated with the other HR response trait

*SNP* single-nucleotide polymorphism, *CHR* Chromosome, *BP* Base pair Position, based on HG build 19, *EA* Effect allele, *EAF* Effect allele frequency from discovery data set, *β* Beta in beats per minute, *SE* Standard Error, *N* effective number of participants, *P* *P*-value.

^a^ Secondary SNP identified (rs2539671) at same locus using conditional analysis

^b^ Secondary SNP identified (rs17180489) at same locus using conditional analysis

**Fig. 2:**
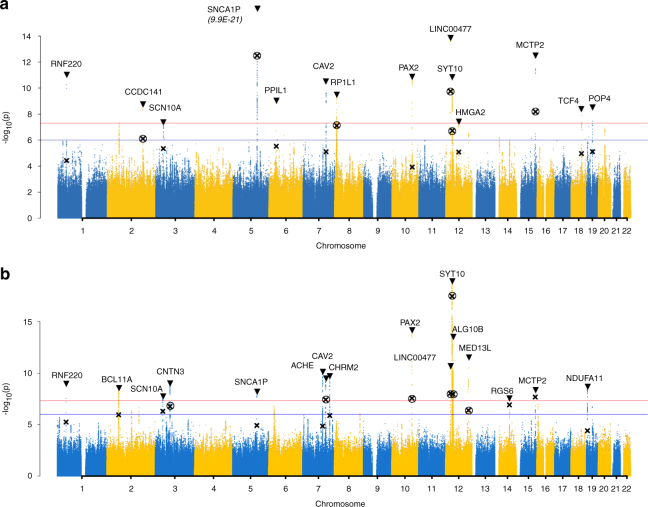
Manhattan plots of ΔHR^ex^ and ΔHR^rec^ in the full cohort analysis. Genome-wide association study for ΔHR^ex^ (**a**) and ΔHR^rec^ (**b**) in 67,257 individuals in the UK Biobank. *P* values, expressed as −log10(P), are plotted according to physical genomic locations by chromosome. Lead SNPs are marked by the triangles. The crosses indicate the *P* values of these SNPs in the discovery data set. Crosses are encircled for SNPs that formally replicated. Locus names of the novel loci correspond to the nearest annotated gene. The blue horizontal line indicates a *P* value threshold of 1 × 10^−6^, corresponding to the lookup significance threshold. The red horizontal line indicates a *P*-value threshold of 5 × 10^−8^, corresponding to genome-wide significance

We next performed a full data set GWAS for each trait (Methods). Manhattan plots including the discovery and full data set GWAS results are shown in Fig. 2 and corresponding QQ plots in Supplementary Fig. 1. Eight additional SNPs reached genome-wide significance for ΔHR^ex^ and 9 SNPs for ΔHR^rec^, respectively, all with concordant directions of effect in the full GWAS data set. All variants were more significant in the full data set than the discovery data set alone. Altogether, across both the replication stage and full data set GWAS, 14 loci were identified for ΔHR^ex^ and 16 loci for ΔHR^rec^ (Tables 1 and 2, Supplementary Tables 3 and 4, Supplementary Fig. 2).

Eight loci (1 Mb regions) were associated with both traits (Tables 1 and 2). Two of the lead SNPs were identical (*RNF220* and *MCTP2*) for both traits, five were in high linkage disequilibrium (LD; *r*^2^≥0.8 at *SCN10A*, *SNCA1P*, *CAV2*, *PAX2* and *LINC00477-SOX5*), thus tagging the same signal, and one variant at *SYT10* was in relatively low LD (with the variant for the other trait (*r*^2^ = 0.32).

Conditional analysis showed evidence for two secondary independent signals, rs2539671 at the *BCL11A* locus and rs17180489 at the *RGS6* locus, associated with ΔHR^rec^ (Table 2, Supplementary Fig. 3). The secondary signal at *BCL11A* was located 147 Kb away from the lead SNP (rs1372876) and it was not in LD with any SNP known to be associated with resting HR or HR variability (HRV, *r*^2^ < 0.1). The secondary signal at the *RGS6* locus was located 41 Kb away from the lead SNP (rs150330648) and has been previously reported to be associated with resting HR^7^.

The heritability estimations (Methods) of ΔHR^ex^ and ΔHR^rec^ were 17.1% and 12.0%, respectively, and their respective genetic correlation was 75.5%. In addition, the 14 newly identified lead SNPs for ΔHR^ex^ explained 0.8% of the trait variance, whereas the 16 lead SNPs and the two secondary signals at the *BCL11A* and *RGS6* loci explained 0.79% of ΔHR^rec^ variance.

Individuals taking beta-blockers were not excluded from our analyses. Sensitivity analysis comparing the *P* values (ρ = 0.93 and ρ = 0.83 for ΔHR^ex^ and ΔHR^rec^, respectively) and the β estimates (ρ = 0.99 and ρ = 0.96 for ΔHR^ex^ and ΔHR^rec^, respectively) from the full GWAS results including or excluding beta-blocker users showed that these were highly correlated, indicating that inclusion of individuals receiving beta-blockers has no relevant effect on our results (Supplementary Table 5).

### Specificity of loci for ΔHR^ex^ and ΔHR^rec^

**Fig. 3:**
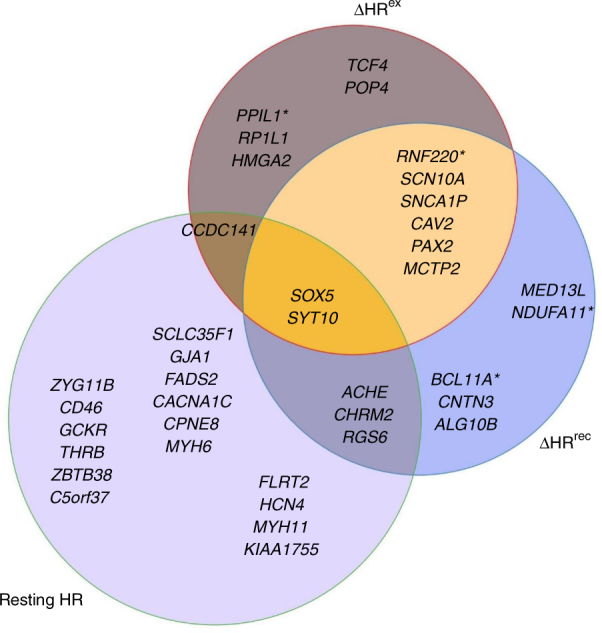
Overlap of loci for ΔHR^ex^, ΔHR^rec^ and resting HR in the full cohort GWAS. The locus names indicate the nearest annotated genes. A substantial number of loci associated with ΔHR^ex^ and ΔHR^rec^ were not associated with resting HR and vice versa. The loci *SOX5* and *SYT10* were identified in all three traits. *: indicates loci that have previously been associated with resting HR but did not reach genome-wide significance in our study

To assess whether the loci identified in the full data set GWAS were specific to ΔHR^ex^ and ΔHR^rec^, we performed an additional GWAS for resting HR in the full cohort (~67,000) and conducted a lookup of lead SNPs identified for the HR response to exercise and to recovery traits. Genetic variants at five loci (*SNCAIP*, *CAV2*, *RP1L1, HMGA2* and *POP4*) for ΔHR^ex^ and at five loci (*BCL11A*, *CNTN3*, *SNCAIP*, *CAV2* and *MED13L*) for ΔHR^rec^ were non-significant for resting HR (*P* > 0.05; loci *SNCAIP* and *CAV2* were in common). These results indicate these SNPs may be specifically associated with HR response traits (Supplementary Table 6). Variants at three loci associated with ΔHR^ex^ (*CCDC141, LINC00477* and *SYT10*) and four loci associated with ΔHR^rec^ (*ACHE*, *CHRM2*, *LINC00477* and *SYT10*) were genome-wide significant for resting HR, with two loci common to both traits (Supplementary Table 6, Fig. 3).

To further explore the relationship and overlap of our discovered loci and those associated with resting HR or HRV, we performed a reciprocal lookup of all published genome-wide significant SNPs^7,9,13^ associated with resting HR (N = 73) and one SNP reported for association with HRV^23^ in the HR response to exercise and to recovery GWAS datasets. Prior work has indicated there is an overlap of loci between resting HR and HRV, with only one variant being solely associated with HRV^23^. Imputed SNPs or good proxies (*r*^2^>0.8) were available for 68/73 SNPs associated with resting HR, and the one HRV variant (Methods). For the ΔHR^ex^ trait, published resting HR SNPs at four loci were genome-wide significant (*SOX5*, *RNF220*, *SYT10* and *PPIL1*), while 25 additional loci were nominally significant (5 × 10^−8^ < *P* < 0.05; Supplementary Data 2). For the ΔHR^rec^ trait, SNPs at seven resting HR loci (*SYT10*, *SOX5*, *UFSP1*, *RNF220*, *ALG10*, *BCL11A* and *CHRM2*) and one SNP associated with HRV at the *NDUFA11* locus were genome-wide significant, while 21 variants were nominally associated (Supplementary Data 3). A lookup of all published resting HR SNPs in our resting HR GWAS results indicated 62/68 SNPs with significant associations (*P* < 0.05), where 15 were genome-wide significant (Supplementary Data 4). The overlap of genome-wide significant loci across resting HR and the HR response to exercise and to recovery traits in our data set is illustrated in Fig. 3.

We observed an overlap of genome-wide significant loci between resting HR and the HR response to exercise and recovery traits. Notably, for ΔHR^ex^ apart from associations with the same SNP or a proxy variant (*r*^2^≥0.8; *n* = 4), there were five additional loci that mapped to the same chromosomal region as a resting HR locus. The lead variant at these five loci was less than 1 Mb away (*CCDC141*, *SCN10A*, *CAV2*, *PAX2* and *SYT10*) from the lead variant of the respective resting HR loci. However, of these five SNPs only the one at the *SYT10* locus was in moderate LD (*r*^2^= 0.62) with the known resting HR SNP, while all other SNPs were in very low LD (*r*^2^ < 0.1) with known resting HR SNPs. For ΔHR^rec^, there were six SNPs associated with a known resting HR variant (*r*^2^≥0.8) and six SNPs mapped to a known resting HR locus based on distance of < 1 Mb. Of these, two SNPs at *CHRM2* and *SYT10* were in moderate LD (*r*^2^>0.57), the four other SNPs at *SCN10A*, *CAV2*, *PAX2* and *RGS6* were in low LD (*r*^2^ < 0.1, Supplementary Table 7).

### Sex-stratified analyses

The clinical course and incidence of many cardiovascular states are well recognised to differ according to gender^24–26^. Related to this, it has been suggested that females have increased parasympathetic and decreased sympathetic control of HR with respect to males^27^. Since the autonomic nervous system plays a key role in the HR response to exercise, we were interested in whether there were sex-based differences in genomic associations with HR response to exercise and recovery traits between males and females. We performed a sex-stratified analysis in the full cohort (*N* = 67,257, with 35,455 females and 31,802 males) for each trait. We identified two additional (i.e., not significantly associated in the primary analysis) new genome-wide significant loci for the ΔHR^ex^ trait. One locus was only significant (*P* < 5 × 10^−8^) in females (*HLA-DRB5/HLA-DRB1*, rs9270779, *P* = 1.20 × 10^−8^) and one in males (*TAF2*, rs60717250, *P* = 2 × 10^−8^, Supplementary Table 8). The identified SNPs at the two loci were not in LD (*r*^2^<0.1) with other loci identified for ΔHR^ex^ or with loci associated with resting HR. We did not observe any sex specific loci for the ΔHR^rec^ trait that were not previously identified in the main analysis (Supplementary Table 8).

### Association of ΔHR^ex^ and ΔHR^rec^ loci with other traits

To explore shared mechanisms of disease, we assessed association of our discovered SNPs (and their proxies, *r*^2^ ≥ 0.8) with other traits from published GWAS (PhenoScanner, see URLs). We observed genome-wide phenotype-genotype associations at four loci for ΔHR^ex^ and four loci for ΔHR^rec^. Three loci (*SCN10A*, *LINC00477-SOX5* and *SYT10*) were common across traits (Supplementary Table 9). The SNPs at *SYT10*, *LINC00477-SOX5* and *SCN10A* were all associated with resting HR and other ECG traits. The SNP associated with ΔHR^ex^ at *HMGA2* was associated with aortic root size and height, and the SNP at *ACHE* for ΔHR^rec^ was associated with resting HR and other ECG traits. The SNP at the *HLA-DRB1/HLA-DRB5* locus associated with ΔHR^ex^ in females was also significantly associated with inflammatory bowel disease and ulcerative colitis.

A lookup of all SNPs associated with HR response to exercise and to recovery (Tables 1 and 2) in the UKB Gene Atlas PheWAS data set, a large database with association results from hundreds of traits in UKB revealed no genome-wide significant associations. One SNP, rs10497529 at *CCDC141* was associated (*P* = 5.24 × 10^−7^) with code I45 conduction disorders.

### Functional annotation of discovered loci

Four lead variants or their close proxies (*r*^2^>0.8) mapping to *CCDC141*, *SCN10A*, *UFSP1* and *NDUFA11* were annotated as missense variants (Tables 1 and 2). Only one variant in *CCDC141* was predicted to be damaging using PolyPhen. This variant has previously been reported to be associated with resting HR^7^. A close proxy (rs372634318, *r*^2^ = 0.86) of the SNP at the *HLA-DRB1/HLA-DRB5* locus associated with ΔHR^ex^ in females only is also a missense variant and is predicted to be damaging using two bioinformatics tools.

### Regulatory variants at ΔHR^ex^ and ΔHR^rec^ loci

As the majority of HR response to exercise and to recovery associated SNPs are non-coding, we also identified regulatory variants, that might affect gene expression levels of their target genes. We interrogated publicly available expression quantitative trait loci (eQTL) datasets through GTEx to highlight potential causal genes and mechanisms at each of the newly identified loci for both HR response traits. The ΔHR^rec^ associated SNP, rs6549649 at *CNTN3* was correlated (*r*^2^ > 0.8) with the top eQTL for *CNTN3* levels in brain nucleus accumbens basal ganglia (*P* = 3.42 × 10^−8^) and spleen (*P* = 1.15 × 10^−8^). The ΔHR^rec^ associated SNP, rs3757868 variant at the *ACHE* locus was associated with expression levels of *ACHE* in the aorta (*P* = 8.37 × 10^−12^) and tibial artery (*P* = 2.04 × 10^−12^) and *SSRT* levels in tibial nerves (*P* = 7.38 × 10^−9^, Supplementary Data 5).

Genetic variants may have a causal effect through regulatory chromatin interactions. We investigated variants at the 30 independent loci associated with ΔHR^ex^ and ΔHR^rec^, as well as those associated with gender specific associations. We identified variants with regulatory potential using RegulomeDB^28^ and found genes whose promoter regions form significant chromatin interaction with them from a range of tissues, we report results from brain, heart and adrenal Hi–C data. We found 32 potential target genes in 17 HR response to exercise and to recovery loci (Supplementary Data 6).

We performed DEPICT analyses to identify enriched pathways and tissues for each HR response to exercise and to recovery trait. We observed no significant results from this analysis. Subsequently, we performed pathway analyses using gprofiler including only candidate genes indicated from eQTL and long-range interaction results (Supplementary Table 10). These analyses indicated enrichment for growth factor response and abnormal autonomic nervous system physiology using ΔHR^ex^ candidate genes and nervous system development and regulation of potassium channel activity using ΔHR^rec^ genes (Supplementary Fig. 4).

We also observed HR response to exercise and to recovery loci to be significantly enriched for DNase I hypersensitive sites (DHSs, Supplementary Fig. 5). We evaluated regions containing all ΔHR^ex^ loci (14 SNPs) and 18 SNPs associated with ΔHR^rec^ including the secondary signals at *BCL11A* and *RGS6*. The highest enrichment for DHSs in ΔHR^ex^ loci was within regions that are transcriptionally active in fetal kidney and fetal renal cortex. For ΔHR^rec^ we observed significant enrichment for DHSs in fetal heart samples.

### Genetic risk score analyses

We created genetic risk scores (GRSs) for each trait to evaluate the impact of the combination of all loci reported here on HR response and cardiovascular mortality risk. We observed individuals in the highest quintile of the distribution of the GRS had a HR response to exercise that was 3.15 (s.e. of 1.15) bpm lower than that of those in the lowest quintile (*P* < 10^−16^). Regarding the GRS for the HR response to recovery, individuals in the highest quintile of the distribution of the GRS had a HR response to recovery that was 10.4 (s.e. of 1.13) bpm lower than that of those in the lowest quintile (*P* < 10^−16^). We did not observe significant associations between the GRSs and cardiovascular mortality risk probably due to a lack of power. There were a total of 118 victims (0.18%) in the full cohort of ~67,000 individuals up to March 2016).

## Discussion

This study systematically investigates the genetic basis of HR response to exercise and recovery using a robust framework including independent discovery and validation samples. Dense HRC imputation yielded a high-quality data set including ~7.8 million variants at minor allele frequencies (MAF)>1% for testing in ~67,000 individuals^29^. The reliability of the phenotypes was examined by analysing raw-ECG recordings to identify and exclude any unreliable automated HR phenotypes before applying genetic analysis.

This strategy allowed us to robustly validate six SNPs associated with HR response to exercise and seven SNPs associated with HR response to recovery. In a GWAS of the full data set, we further identified eight and nine loci for each trait, respectively. In total, 30 loci are reported therein and two additional loci from sex-stratified analyses. In fact, our findings from the sex-stratified analyses confirm that there are gender differences in the genetic architecture of HR response to exercise and to recovery, supporting conclusions from previous studies^24–27^.

Eight loci were common to both HR response to exercise and recovery traits (Fig. 3). This was expected due to the relatively high genotypic correlation between them (ρ = 0.75). The overlap of loci between the HR response to exercise and to recovery traits and resting HR in our study was smaller (6 loci, Fig. 3), probably due to the lower phenotypic and genotypic correlations between each trait with resting HR (phenotypic correlation of ρ = −0.30 and genotypic correlation of ρ = 0.41 for both traits). Small effect sizes were observed for all SNPs reported in this study, having similar magnitudes as variants previously associated with resting HR (0.12–0.63 bpm per allele)^7^ (Tables 1 and 2).

In our data set, the heritability of resting HR, HR response to exercise and to recovery was 18%, 17% and 12%, respectively, suggesting that the response of HR to exercise and to recovery is complex and largely affected by environmental contributions like the resting HR. A recent study including ~150,000 individuals in UKB has estimated a similar heritability of resting HR (21% using BOLT-REML^7^), which suggests that our calculations for exercise and recovery traits were sufficiently powered.

Bioinformatics analyses indicated several candidate genes at loci specifically associated with HR response to exercise, recovery or with both traits in our data set. We highlight the function of four genes. For HR response to exercise, a potential candidate gene is *BTB* (broad complex, tramtrack and bric à brac) Domain Containing 9 (*BTBD9*). Although its specific function is difficult to ascertain due to its ubiquitous expression, it has been shown that *BTB* proteins play a role in synaptic plasticity and neurotransmission^30^. Notably, *BTBD9* is among pathways related to the regulation of the circadian rhythm^31^, which is known to be involved in cardiac parasympathetic modulation^32^. Further evidence of the involvement of *BTBD9* in neurophysiology has been suggested in other studies including disruption of sleep and motor activity^33^.

Three new candidates are highlighted for the HR response to recovery. *CNTN3*, which encodes Contactin-3 or BIG1, is part of a subgroup of molecules belonging to the immunoglobulin superfamily that are expressed exclusively in the nervous system^34^. At present, *CNTN3* expression on the cardiac neural axis is unknown. However, the SNP associated with ΔHR^rec^ was significantly associated with changes in expression levels of *CNTN3* in the nucleus accumbens (basal ganglia). Specifically, the allele associated with a decrease in ΔHR^rec^ was associated with increased expression of *CNTN3*. Since reduced HR recovery is likely to be a reflection of decreased vagal activity^35^, our work suggests that *CNTN3* may also be relevant to cardiac parasympathetic modulation. A second candidate gene indicated is the Ca^2+^-dependent activator protein for secretion 1 (*CAPS1*) gene at the *FUT5* locus. *CAPS1* is present in neurones and endocrine cells, and is involved in mediating exocytosis from large dense-core vesicles^36^. This is particularly relevant in the adrenal medulla where it has been shown to affect catecholamine release^37^. It is therefore plausible that the association between *CAPS1* and increased HR response to recovery is mediated by the sympathetic nervous system. A third candidate gene is *ALG10B*, which has been linked with the K(+) channel regulatory protein *KCR1*^38^, may regulate cardiac automaticity^39^. Consequently, *ALG10B* could be important in reducing the heart rate during recovery.

The bioinformatics and pathway analysis highlight the role of a number of genes linked to autonomic nervous system activity and cellular electrophysiology. It is recognised that HR response to exercise is driven by an initial parasympathetic withdrawal and then an increased sympathetic neural drive acting directly on the sino-atrial node. This process involves baro-reflex resetting and feedback from muscle mechanoreceptors^40^.

HR response to recovery is driven by a sympathetic withdrawal and a parasympathetic reactivation to slow HR. It is interesting to note that the DNase I hypersensitivity enrichment in renal tissue identified by the FORGE analysis was associated with fetal kidney and renal cortical tissue. This is where renin secreting juxtaglomerular cells reside, these determine angiotensin II levels and systemic vascular resistance, and may modify sympathetic activity and hence HR as a result of baro-receptor reflex effects. Our pathway analysis results suggest that HR might be modified by an increased potassium channel activity during repolarization. This would allow the adaptation of the action potential duration to changes in HR. In addition, the HR could also be reduced by the activation of potassium currents around the resting membrane potential that affect the pacemaking diastolic depolarisation in sinoatrial cells. Thus, genes modulating both autonomic activity and cardiac cellular electrophysiology, may play key roles in HR responses to exercise and to recovery.

We did not observe any significant association between our GRSs and cardiovascular mortality risk. This was in part expected due to the design of the UKB study to recruit relatively healthy individuals aged 40–69 years and the low number of victims at the moment in this study^41^. However, we did observe that individuals in the highest quintile of the GRSs had a significantly reduced capacity to adapt their HR to exercise and to recovery, both indicators of cardiovascular mortality risk. These results are encouraging, and we expect the lack of significant associations between the GRSs and cardiovascular mortality risk might be due to an issue of power, due to the reduced number of subjects in the risk group and to the limited number of identified variants. Future studies will permit expansion of these analyses in the full (N~500,000) UKB cohort and in other datasets.

Our study has some limitations. Although we have utilised data from one of the largest datasets with HR measured both during exercise and recovery stages, we identified a total of 30 SNPs, of which 13 formally replicated. Although the remaining identified SNPs were genome-wide significant, they still require formal replication in an independent data set. The relatively small sample size available with these phenotypes might have also hampered the discovery of additional loci that could contribute to the heritability of these traits. Another potential limitation is that the adjustment for resting HR might have underpowered the identification of genetic signals with a similar impact on resting HR and on HR response to exercise and to recovery traits. Finally, the test was designed in a way that participants were at their sub-maximal effort. Therefore, participants did not reach their maximum HR during the test and this might have limited the quantitative span of our HR response to exercise and to recovery traits.

Whilst our paper was under consideration a study investigating HR response to exercise and recovery traits was published^21^. Verweij et al performed a GWAS in ~ 50 k individuals using ECG derived HR measurements in UKB. They identified 25 SNPs at 23 independent loci, with a *P*-value threshold of 8.3 × 10^−9^. As expected, there is overlap between our findings and theirs: we report 16 of their 23 loci. There are seven loci which were not significantly associated in our analyses with either of our traits, and we identified six loci that were not reported in their study. The dissimilarities in findings may in part be explained by the differences in our phenotype definitions. Although the trait of HR response to exercise is similar in theory, Verweij et al.^21^ calculated five HR response to recovery traits; of these, one (HRR50—the response of HR 50 s after peak) would be the comparable trait to HR response to recovery in our study. Other possible reasons for the differences may include sample size; ours was slightly larger as we used validated UKB HR measurements together with our in-house derived ECG HR measurements.

In summary, our findings reinforce current understanding of autonomic response to exercise and to recovery and will guide future studies evaluating its contribution in cardiovascular risk prediction as well as identifying new therapeutic targets to modulate heart rate response to exercise and to recovery.

## Methods

### UK Biobank

UKB is a prospective study of 500,000 volunteers, comprising relatively even numbers of men and women aged 40–69 years old at recruitment, with extensive baseline and follow-up clinical, biochemical, genetic and outcome measures. The UKB study has approval from the North West Multi-Centre Research Ethics Committee, and all participants provided informed consent.

Genotyping was performed by UKB using the Applied Biosystems UK BiLEVE Axiom Array or the UKB AxiomTM Array^41^. SNPs were imputed centrally by UKB using the Haplotype Reference Consortium (HRC) panel. Information on UKB array design and protocols is available on the UKB website (URLs).

### Exercise test protocol and data acquisition

The exercise test started with 15 s of rest (pre-test), followed by 6 min of exercise (cycling) initially at constant load (2 min), then at increasing workload (4 min), and a 1 min recovery period without pedalling. Automatic HR measurements were taken throughout the protocol and were provided by UKB, together with the raw ECG recordings^42^.

Approximately 95,000 individuals participated in the exercise test using a stationary bicycle in conjunction with a 4-lead electrocardiograph device at the initial assessment (2006–2008), from which ~20,000 individuals were invited to the first repeat assessment in 2011–2013.

### Selection of individuals for analysis

An overview of the process used to select individuals who participated in the exercise test for subsequent genetic analysis is presented in Supplementary Fig. 6. From the ~95,000 individuals invited, 79,745 completed the test. Automated HR measurements provided by UKB (UKB HR measurements) were available in 78,655 individuals. If available, data from the test performed at initial assessment (63,972 individuals) were analysed; otherwise, data from the first repeat assessment (14,683 individuals) were included in the analysis.

Validation of UKB HR measurements was performed by comparing them with traits derived from available raw ECG recordings (N = 62,272; ECG HR measurements) using our bespoke algorithms^43^. ECGs were selected for visual inspection when ECG and UKB HR measurements did not match for resting, peak HR or recovery HR. The visual inspection was to ensure whether the UKB HR values could be trusted. UKB HR measurements were rejected if the ECG recordings contained episodes without clear identifiable QRS complexes. In other cases the mismatch in HR values was caused by failure of the algorithm to detect QRS complexes. Detection of QRS complexes was then corrected by hand and the UKB HR values were accepted for analysis. Correlation for resting HR, maximum exercise and recovery was >0.9 (Supplementary Fig. 7). After visual inspection, 1,482 individuals were excluded. In 16,383 individuals’ validation was not possible due to the absence of raw ECG recordings. However, the statistical distribution of UKB HR measurements at rest, maximum exercise and recovery for these individuals closely matched that of the validated measurements (Supplementary Table 11) and traits from these individuals were included in the analysis. In total 77,173 individuals with reliable UKB HR measurements at three different phases of the exercise test were used to derive our phenotypes of interest.

### Genetic and phenotypic QC

Genetic quality control (QC) was performed on the set of individuals who participated in the exercise test protocol (*N* = 95,216). Individuals with bad genotype quality, provided by UKB, i.e. high missingness or heterozygosity (*N* = 1472) and discordance between the self-reported sex and the sex inferred from the genotypes (*N* = 982) were excluded^41^. We then restricted our data set to individuals of European ancestry only (*N* = 85,522).

We used the k-means function in R as a clustering algorithm, to objectively and statistically select the clusters according to information from PC1 and PC2. The k-means algorithm ‘partitions the points into *k* groups such that the sum of squares from points to the assigned cluster centres is minimised.’ Then, we applied k-means separately to cluster according to each of PC1 and PC2, and initially only with *k* = 4, for a 4-way clustering, to correspond to the 4 main ethnic clusters within UKB: White, African, Asian and Chinese.

We then created an overall clustering, according to the intersections of the PC1-4means-clustering and the PC2-4means-clustering, so that participants were only categorised as ‘White’ overall, if they were contained in the ‘White’ cluster for both PC1 and PC2. Next, we created an overall ‘Mixed/Other’ cluster, for any participants, whose clustering differed between PC1 and PC2. Finally, we combined the PCA ancestry clusters with the self-reported ethnicity. Individuals were only included if the results PCA-clustering results matched the self-reported ancestry. However, we count ‘mixed’, ‘other’ and ‘missing’ as being broad/uncertain self-reported ethnicity, which have now been validated more objectively from the genetic PCA data. Over the 77,173 individuals for whom it was possible to derive the phenotypes, 69,353 complied with genetic QC and were of European ancestry (Supplementary Fig. 8).

Before genetic analysis, we further excluded individuals (*N* = 2,317) based on existing medical conditions known to affect HR (namely atrial fibrillation, history of myocardial infarction or heart failure, (supra)-ventricular tachycardia, atrio-ventricular nodal re-entrant tachycardia, second or third degree atrioventricular block, and use of a pacemaker) and/or individuals on HR altering medications (non-dihydropyridine calcium antagonists; Anatomic Therapeutic Chemical (ATC) code C08D; digoxin (ATC code C01AA5), and amiodarone (ATC code C01BD01; Supplementary Fig. 8).

Individuals with extreme UKB HR measurements at rest (<40 or >120 bpm), peak exercise (>200 bpm) or at 1 min post-exercise (<40 or >200 bpm) were also excluded. This led to *N* = 66,800 individuals with ΔHR^ex^ measurements and *N* = 66,665 individuals with ΔHR^rec^ measurements for analysis. A total of *N* = 66,844 individuals with resting HR measurements were also available for analysis (Supplementary Fig. 8).

### Derivation of ΔHR^ex^ and ΔHR^rec^

HR response to exercise (ΔHR^ex^) and HR response to recovery (ΔHR^rec^) were computed from the resting HR, peak HR and recovery HR, where resting HR was defined as the mean HR during pre-test period, peak HR as the maximum HR during exercise, and recovery HR as the minimum HR 1 min after the peak exercise. The phenotypic traits Δ*HR*^*ex*^ and Δ*HR*^*rec*^ were then computed as:

$$\Delta \mathrm{HR}^{\mathrm{ex}} = {\mathrm{Peak}}\,{\mathrm{HR}} - \,{\mathrm{Resting}}\,{\mathrm{HR}}$$

$$\Delta \mathrm{HR}^{\mathrm{rec}} = {\mathrm{Peak}}\,{\mathrm{HR}} - {\mathrm{Recovery}}\,{\mathrm{HR}}$$

Inverse-normal transformation of the traits was not performed since their distribution approximated a normal distribution (Supplementary Fig. 9).

### Genetic analyses

As we did not have access to an independent study with raw ECG recordings during exercise and genetic data that could serve as a replication study, and with the limited sample size we randomly divided our cleaned data set into discovery (*N* ~ 40,000) and replication (*N* ~ 27,000) datasets (Fig. 1). For the discovery data set, we selected the model SNPs from the genotyped SNPs, required for the subsequent GWASs using PLINK 1.9^44^. This selection was based on the following criteria: a minor allele frequency (MAF) > 1%, a Hardy–Weinberg equilibrium (HWE) with a threshold of *P*-value = 1 × 10^−6^, and missingness < 0.0015.

Then, we estimated the proportion of ΔHR^ex^ and ΔHR^rec^ explained by additive genetic variation (heritability) using a variance components method (BOLT-REML)^45^, with the model SNPs and ~ 7.8 million imputed variants with MAF ≥ 1%, imputation quality (INFO) > 0.3 using the full data set.

Next, we performed a GWAS for each trait using a linear mixed model method (BOLT-LMM)^46^ under the additive genetic model including ~ 7.8 million imputed SNPs with MAF ≥ 1% and INFO > 0.3. We used the heritability estimation obtained from BOLT-REML.

For ΔHR^ex^ and ΔHR^ex^ traits we included the following covariates: sex, age, body mass index (BMI), resting HR, resting HR^2^ and a binary indicator variable for the genotyping array (UK Biobank versus UK BiLEVE).

Although BOLT-LMM software accounted for genetic relatedness within the analysed cohort, individuals across discovery and replication cohorts could be related, making both datasets not completely independent from each other. Therefore we removed a total of N = 818 first and second degree related individuals (kinship coefficient > 0.88) from the replication cohort as indicated from UK Biobank^41^.

We also performed a GWAS for both traits in the replication data set following removal of related individuals using the heritability parameter estimated in the discovery data set.

All of our genetic analysis in UKB were restricted to variants imputed using the HRC panel^41^.

### Replication analyses

All SNPs with *P* < 1 × 10^−6^ from the discovery analysis for both traits were compiled and SNPs were mapped to individual loci based on genomic distance of >500 Kb to each side of another SNP. If multiple SNPs fit the selection criteria for a single region, only the SNP with the smallest *P* value was considered for follow-up. As a QC step, we reviewed each selected SNP to check for unrealistically high beta values, large standard errors, and none were observed. Locus Zoom plots were produced for all selected SNPs and these were carefully reviewed. Fourteen variants for ΔHR^ex^ and 17 variants for ΔHR^rec^ were taken forward into replication. Replication was confirmed if *P* (one-tailed) ≤0.05/14 = 2.60 × 10^−3^ for ΔHR^ex^ and ≤0.05/16 = 3.10 × 10^−3^ for ΔHR^rec^ and the effect (β) was in the direction observed in discovery analyses for each trait in the replication cohort.

### Full data set analyses

We also performed a full data set GWAS for each trait using BOLT-LMM. Additional loci for each trait reaching a genome-wide significance threshold (*P* ≤ 5 × 10^−8^) from the full data set GWAS are reported.

We additionally performed a GWAS for resting HR in the full data set, *N* = 67,257 to serve as a reference for interpreting results from our traits. The regression model included the following covariates: sex, age, BMI and a binary indicator variable for UKB versus UK BiLEVE genotyping arrays.

### Conditional analyses

To examine the existence of independent SNPs to lead SNPs, we applied genome-wide complex trait analysis (GCTA)^47^ for all validated and genome-wide significant loci from the full data set GWAS. A secondary signal would be declared if: (i) the newly identified SNP original *P* value was lower than 1 × 10^−6^; (ii) there was less than a 1.5-fold difference between the lead SNP and secondary association *P* values on a –log_10_ scale, i.e., if –log_10_(*P*_*lead*_)/-log_10_(*P*_sec_) < 1.5; and (iii) if there was less than a 1.5-fold difference between the main association and conditional association *P* values on a –log_10_ scale, i.e., if –log_10_(*P*_*sec*_)/-log_10_(*P*_cond_) < 1.5.

### Percent variance

The percent of variance explained in ΔHR^ex^ and ΔHR^rec^ by all genome-wide significant variants and the secondary signal for ΔHR^rec^ (*N* = 14 and *N* = 18, respectively) was calculated using standard regression models including analysis covariates (see above) and each SNP separately for each trait. Each trait, ΔHR^ex^ and ΔHR^rec^, was regressed initially only on analysis covariates and then on covariates and the respective SNPs. Both *r*^2^ values obtained from these two regressions were used as estimations of the percent variance explained by the respective models. Through subtraction of the both *r*^2^ values, we determined the percent variance explained by all newly discovered SNPs.

### Sensitivity analyses

Previous GWAS and Exome-chip analyses for resting HR have shown that use of beta-blockers do not significantly affect the effect sizes of associated variants^9^. However, since the traits analysed in study might not only depend on resting HR, we undertook additional association analyses excluding individuals taking beta-blockers for our reported SNPs using the full data set. We then calculated the Spearman’s correlation coefficient between the β estimates and *P* values for the results including and excluding individuals under beta-blockers for each trait.

### Sex-stratified analyses

For each trait, we performed a GWAS for men and women separately in the full cohort including the same covariates in the regression model as specified above, but excluding sex.

### Bioinformatics analyses

We performed several analyses to annotate the HR response to exercise and recovery associated SNPs, at the variant and gene level (all SNPs in LD *r*^2^≥0.8 with the HR response to exercise and to recovery associated SNPs were considered). LD was calculated using genetic data from UKB if the lead SNP was imputed using the HRC reference panel in order to calculate pairwise-LD for all associated SNPs. For SNPs not available in UKB, we used the 1000 Genomes Project phase 3 (1000 G) reference panel. The *r*^2^ of pairwise SNPs (minimum *r*^2^ = 0.8 and maximum distance between a pair of SNPs is 1 Mb) were computed using PLINK^44^.

Using University of California, Santa Cruz (UCSC) known genes, we annotated each lead SNP with the nearest genes and those found within 5 kb. We used VEP^48^ to characterise the variants, including the impact of amino acid substitutions based on a range of prediction tools including SIFT and PolyPhen-2 and we also assessed the conservation scores. Missense variants were annotated to be damaging if indicated by two or more methods.

We evaluated all SNPs in LD (*r*^2^≥0.8) with our validated lead SNPs to explore if there was support for mediation of eQTLs in 44 tissues in the GTEx database. We also sought to identify if the validated variant at each locus was coincident with the the strongest evidence of eQTL association for that gene, and we focussed on reporting results from the brain, heart and adrenal tissue.

We identify potential target genes of regulatory SNPs using long-range chromatin interaction (Hi–C) data from left and right ventricles, adrenal glands, neural progenitor stem cells, hippocampus and cortex^49^ which tissues and cell types are considered relevant for regulating heart rate. Hi–C data is corrected for genomic biases and distance using the Hi–C Pro and Fit-Hi-C pipelines according to Schmitt et al.^49^ (40 kb resolution—correction applied to interactions with 50 kb-5 Mb span). We find the most significant promoter interactions for all potential regulatory SNPs (RegulomeDB score ≤5) in LD (*r*^2^ ≥ 0.8) with our sentinel SNPs and choose the interactors with the SNPs of highest regulatory potential to annotate the loci.

We also performed enrichment testing across all loci. We used DEPICT^50^ (Data-driven Expression-Prioritised Integration for Complex Traits) to identify cells and tissues in which the HR response to exercise and to recovery loci were highly expressed. Subsequently, for the best candidate genes per locus (Supplementary Table 10), we used g:profiler that performs functional profiling of gene lists using various kinds of biological evidence (including GO, HPO annotation). Enrichments with FDR < 0.05 were deemed significant.

Furthermore, to investigate regulatory regions, we used FORGE to investigate cell-type-specific enrichment within DNase I-hypersensitive sites in 123 cell types from ENCODE and the Epigenomics Roadmap Project^51^. Validated lead SNPs from our study were analysed along with independent secondary signals (with *P* < 10^−6^) to evaluate the overall tissue-specific enrichment of the HR response to exercise and to recovery variants. Our directly our curated candidate regulatory SNPs were checked for overlap with cell-type-specific DNase I-hypersensitive signals in a second analysis which did not include an LD filter.

We queried associated SNPs against PhenoScanner^52^ to investigate trait pleiotropy, extracting all association results with a nominal significance of *P* *<* 0.05 for full reporting, and then extracted genome-wide significant results to highlight loci with the strongest evidence of association with other traits and we also indicate results with *P* *<* 1 × 10^−4^. We also accessed results from GeneAtlas to determine further phenotypic associations of our associated variants. We next performed an extensive review of all highlighted candidate genes from bioinformatics analyses at the 31 loci. The National Center for Biotechnology Information (NCBI) Gene database and GeneCards®: The Human Gene Database were used to obtain official full names and, where relevant, common aliases for each candidate gene product. NCBI’s PubMed was used to interrogate primary literature pertaining to gene function. We also reviewed gene-specific animal models using International Mouse Phenotyping Consortium and the Mouse Genome Informatics database.

### Genetic risk scores

We constructed GRSs in the full data set by aggregating all lead and secondary SNPs separately for each trait using the beta estimates from the replication GWASs as independent, unbiased weights, to assess the combined effect of the identified variants on each trait, respectively, and cardiovascular mortality risk, while avoiding bias from ‘winner’s curse’.

The GRS for each trait was standardised to have a mean of 0 and a standard deviation of 1. We then assessed the association of the continuous GRS variable with each HR response trait by simple linear regression adjusting for the same covariates used in the genetic analyses and for the ten first principal components. Related individuals were also excluded. We also ran logistic regression to examine the association of the GRSs with cardiovascular mortality risk. We then compared HR response levels and cardiovascular mortality risk for individuals in the top (highest quintile) and bottom (lowest quintile) 20% of the GRSs distributions.

### URLs

For UK Biobank, www.ukbiobank.ac.uk; for Haplotype Reference Consortium panel, http://www.haplotype-reference-consortium.org/site; for National Center for Biotechnology Information (NCBI) Gene database, see http://www.ncbi.nlm.nih.gov/gene/; for NCBI’s PubMed, http://www.ncbi.nlm.nih.gov/pubmed/; for International Mouse Phenotyping Consortium, http://www.mousephenotype.org/; for Mouse Genome Informatics, http://www.informatics.jax.org; for Gene Atlas PheWas, http://geneatlas.roslin.ed.ac.uk; for The Human Gene Database, http://v4.genecards.org/. For FORGE, http://browser.1000genomes.org/Homo_sapiens/UserData/Forge?db=core. For GTEx, www.gtexportal.org; for Phenoscanner, http://www.phenoscanner.medschl.cam.ac.uk; for RegulomeDB, http://www.regulomedb.org.

### Data availability

The HR data generated during the current study are available from the UK Biobank data repository, which can be accessed by researchers upon application. These data include the GWAS summary data for the HR responses to exercise traits and resting HR. The genetic and phenotypic UK Biobank data are also available upon application to the UK Biobank. All replication data generated during this study are included in the published article and are contained within the supplementary tables provided.

## Electronic supplementary material

Supplementary Information

Description of Additional Supplementary Files

Supplementary Data 1

Supplementary Data 2

Supplementary Data 3

Supplementary Data 4

Supplementary Data 5

Supplementary Data 6
