# Supplementary material for: Thirty loci identified for heart rate response to exercise and recovery implicate autonomic nervous system
Source: Nat Commun. 2018 May 16;9:1947. doi: 10.1038/s41467-018-04148-1 (PMC5955978; doi:10.1038/s41467-018-04148-1)
Supplement: Supplementary file 1 — Supplementary Information [file 41467_2018_4148_MOESM1_ESM.pdf]

Ramírez et al. Thirty loci identified for heart rate response to exercise and recovery implicate the autonomic nervous system

Supplementary Information File

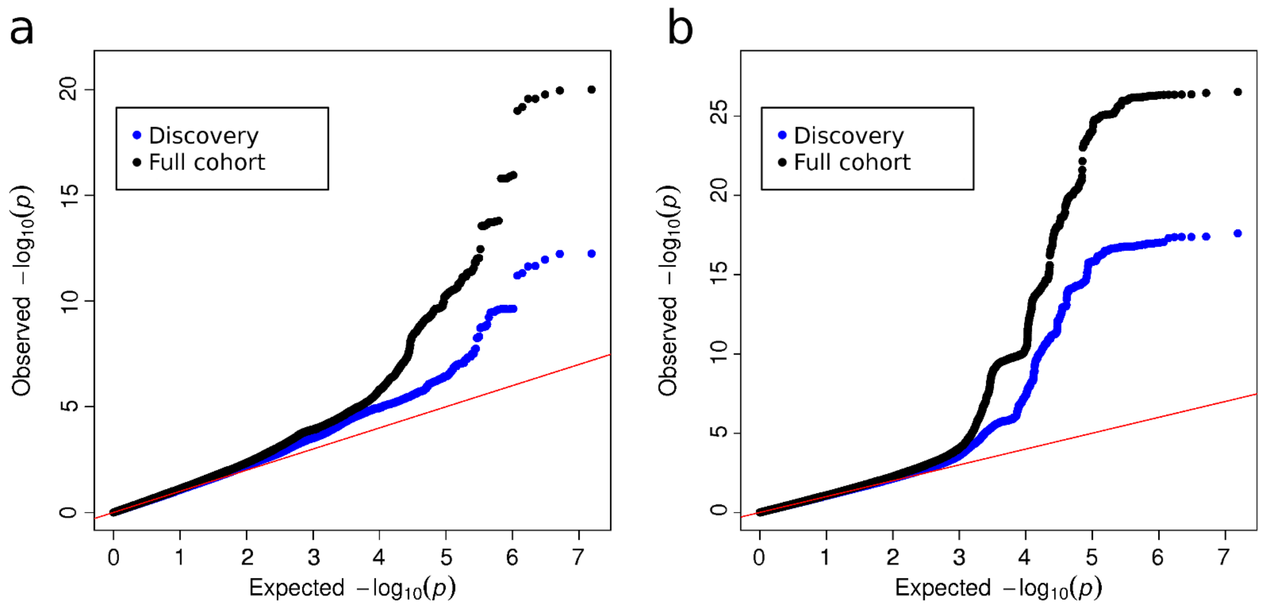

Supplementary Figure 1: QQ plots for HR response to exercise and recovery in the discovery and full cohort. a, HR response to exercise (ΔHR^{ex}) in the discovery (blue) and full cohort analysis (black) data. **b**, HR response to recovery (ΔHR^{rec}). Corresponding λ values for ΔHR^{ex} are 1.099 and 1.15 for discovery data and full cohort data respectively, and 1.05 and 1.099 for ΔHR^{rec}.

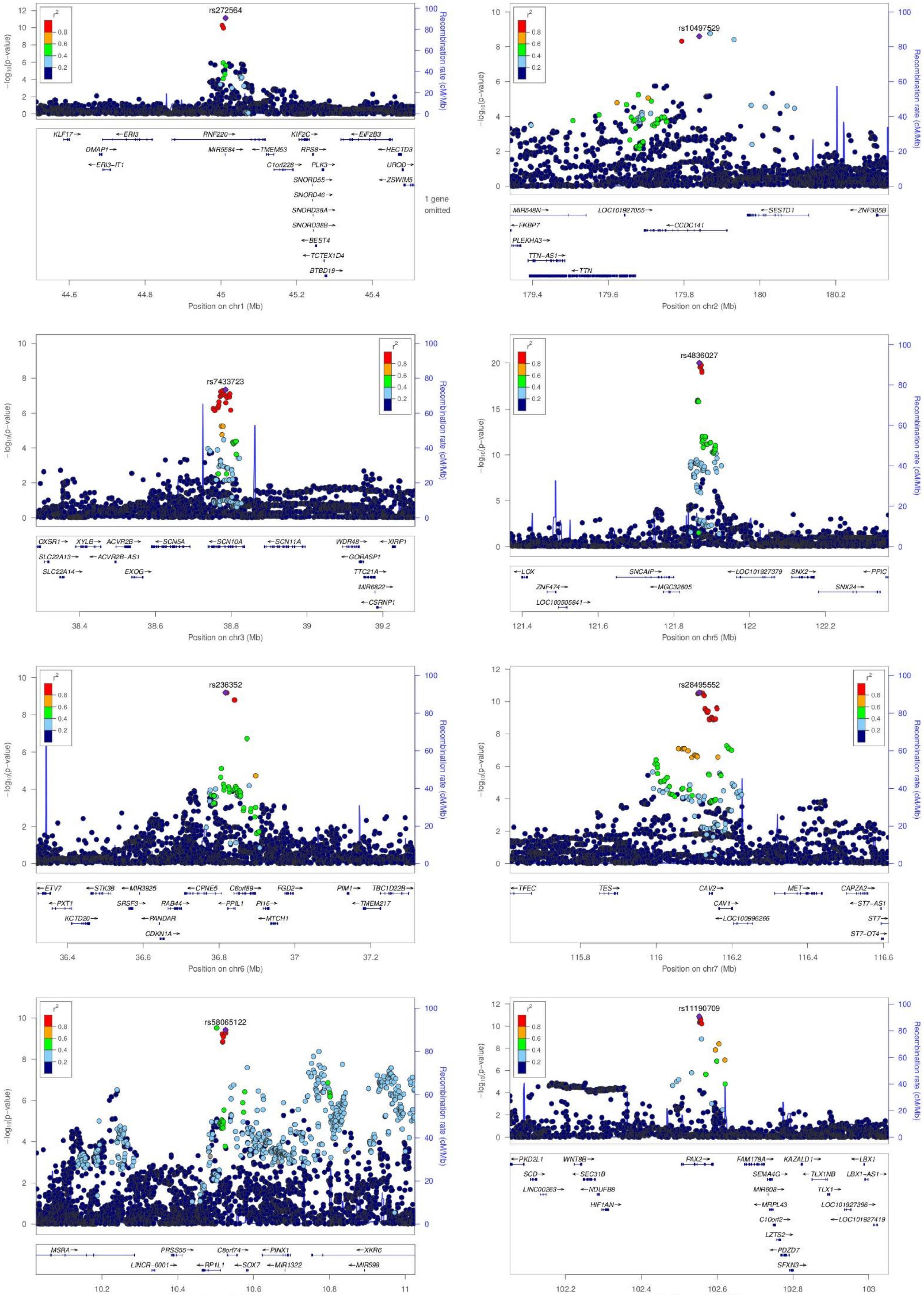

rs272564
−log10(p−value)
Recombination rate (cM/Mb)
Position on chr1 (Mb)
KLF17 ERI3 RNF220 KIF2C EIF2B3
DMAP1 MIR5584 TMEM53 RPS8 HECTD3
ERI3−IT1 C1orf228 PLK3 UROD
SNORD55 ZSWIM5
SNORD46
SNORD38A
SNORD38B
BEST4
TCTEX1D4
BTBD19
1 gene omitted
rs10497529
Position on chr2 (Mb)
MIR548N LOC101927055 SESTD1 ZNF385B
FKBP7 CCDC141
PLEKHA3
TTN−AS1
TTN
rs7433723
Position on chr3 (Mb)
OXSR1 XYLB ACVR2B SCN5A SCN10A SCN11A WDR48 XIRP1
SLC22A13 ACVR2B−AS1 GORASP1
SLC22A14 EXOG TTC21A
MIR6822
CSRNP1
rs4836027
Position on chr5 (Mb)
LOX SNCAIP LOC101927379 SNX2 PPIC
ZNF474 MGC32805 SNX24
LOC100505841
rs236352
Position on chr6 (Mb)
ETV7 STK38 MIR3925 CPNE5 C6orf89 FGD2 PIM1 TBC1D22B
PXT1 SRSF3 RAB44 PPIL1 PI16 TMEM217
KCTD20 PANDAR MTCH1
CDKN1A
rs28495552
Position on chr7 (Mb)
TFEC TES CAV2 MET CAPZA2
CAV1 ST7−AS1
LOC100996266 ST7
ST7−OT4
rs58065122
Position on chr8 (Mb)
MSRA PRSS55 C8orf74 PINX1 XKR6
LINCR−0001 RP1L1 SOX7 MIR1322 MIR598
rs11190709
Position on chr10 (Mb)
PKD2L1 WNT8B PAX2 FAM178A KAZALD1 LBX1
SCD SEC31B SEMA4G TLX1NB LBX1−AS1
LINC00263 NDUFB8 MIR608 TLX1
HIF1AN MRPL43 LOC101927396
C10orf2 LOC101927419
LZTS2
PDZD7
SFXN3

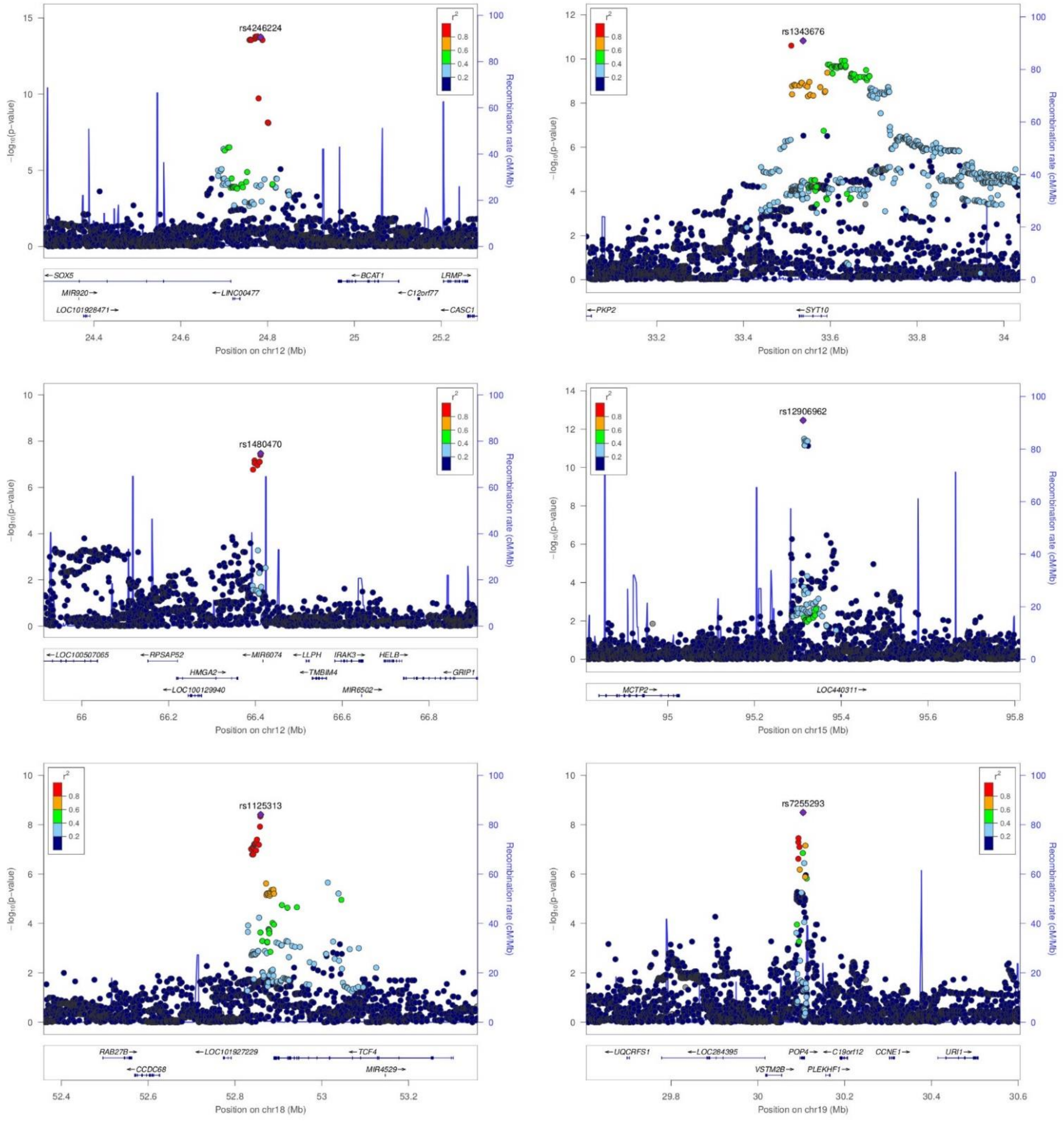

Supplementary Figure 2a: LocusZoom plots for the novel loci for HR response to exercise, $\Delta\mathrm{HR}^{\mathrm{ex}}$, in the full cohort data.

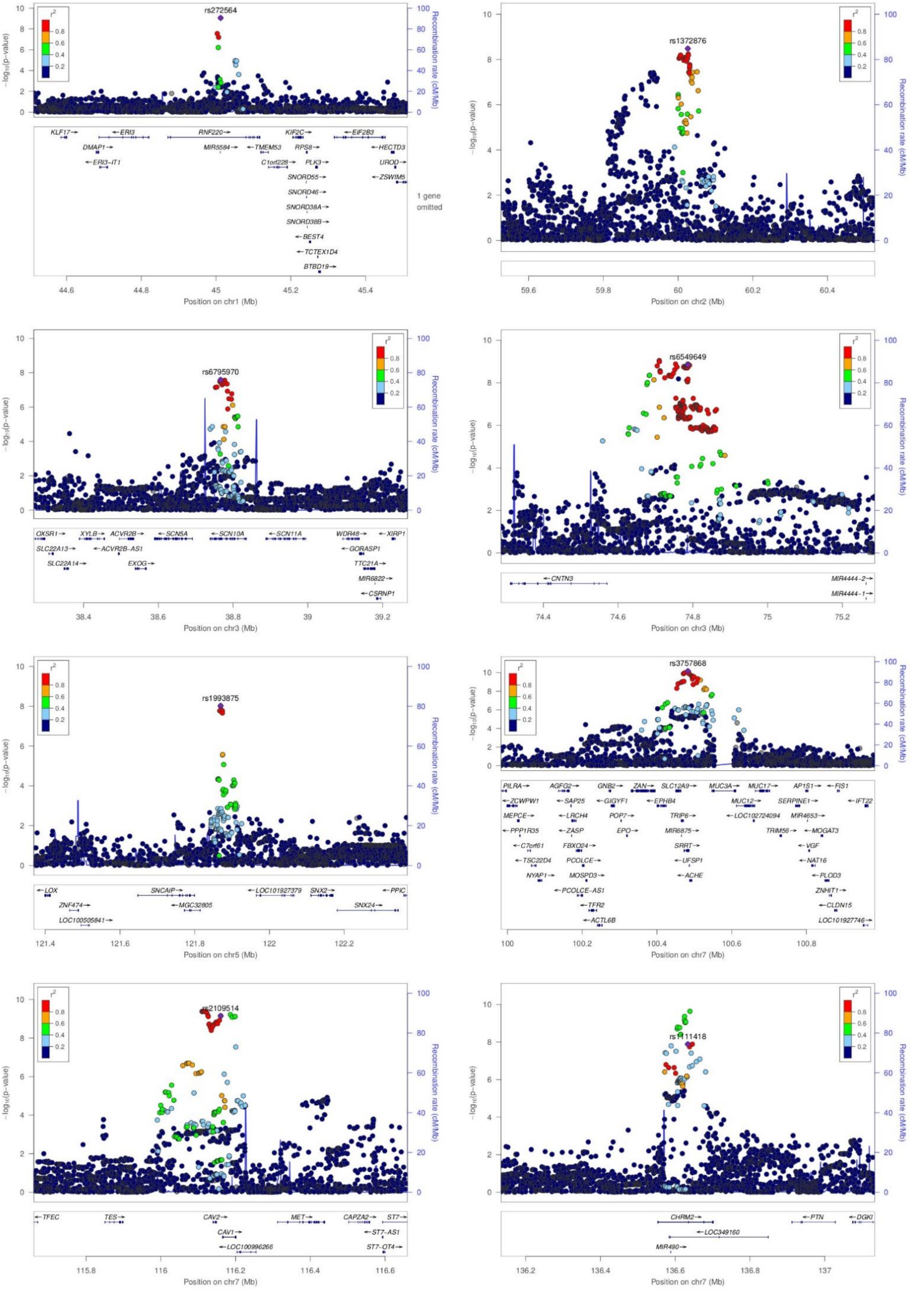

rs272564
Position on chr1 (Mb)
KLF17 ERI3 RNF220 KIF2C EIF2B3 DMAP1 MIR5584 TMEM53 RPS8 HECTD3 ERI3-IT1 C1orf228 PLK3 UROD ZSWIM5 SNORD55 SNORD46 SNORD38A SNORD38B BEST4 TCTEX1D4 BTBD19
1 gene omitted
rs1372876
Position on chr2 (Mb)
rs6795970
Position on chr3 (Mb)
OXSR1 XYLB ACVR2B SCN5A SCN10A SCN11A WDR48 XIRP1 SLC22A13 ACVR2B-AS1 GORASP1 SLC22A14 EXOG TTC21A MIR6822 CSRNP1
rs6549649
Position on chr3 (Mb)
CNTN3 MIR4444-2 MIR4444-1
rs1993875
Position on chr5 (Mb)
LOX SNCAIP LOC101927379 SNX2 PPIC ZNF474 MGC32805 SNX24 LOC100505841
rs3757868
Position on chr7 (Mb)
PILRA AGFG2 GNB2 ZAN SLC12A9 MUC3A MUC17 AP1S1 FIS1 ZCWPW1 SAP25 GIGYF1 EPHB4 MUC12 SERPINE1 IFT22 MEPCE LRCH4 POP7 TRIP6 LOC102724094 MIR4653 PPP1R35 ZASP EPO MIR6875 TRIM56 MOGAT3 C7orf61 FBXO24 SRRT VGF TSC22D4 PCOLCE UFSP1 NAT16 NYAP1 MOSPD3 ACHE PLOD3 PCOLCE-AS1 ZNHIT1 TFR2 CLDN15 ACTL6B LOC101927746
rs2109514
Position on chr7 (Mb)
TFEC TES CAV2 MET CAPZA2 ST7 CAV1 ST7-AS1 LOC100996266 ST7-OT4
rs1111418
Position on chr7 (Mb)
CHRM2 PTN DGKI LOC349160 MIR490
−log10(p-value)
Recombination rate (cM/Mb)
r²

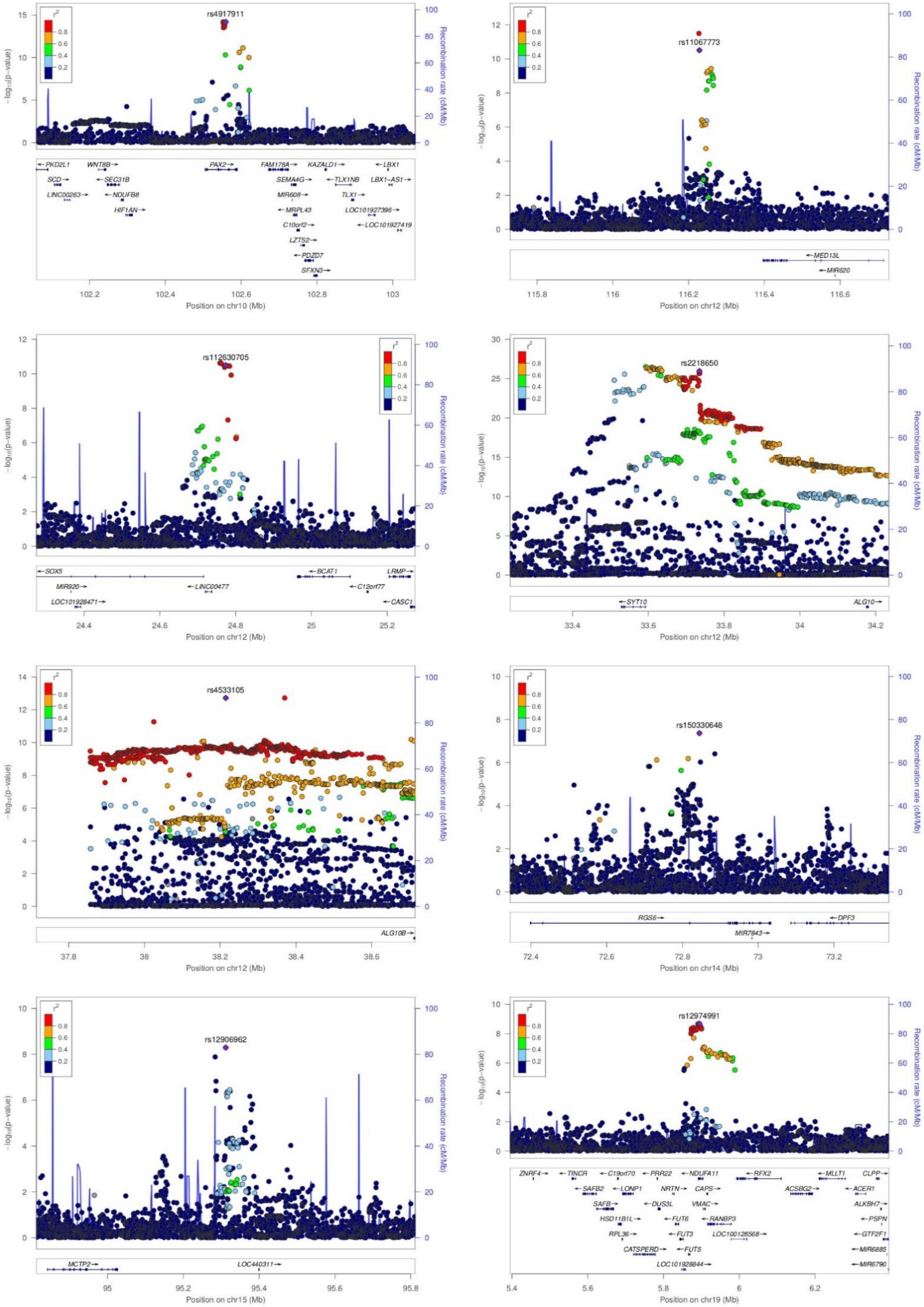

rs4917911
-log10(p-value)
Recombination rate (cM/Mb)
r2
0.8
0.6
0.4
0.2
PKD2L1
WNT8B
SCD
SEC31B
LINC00263
NDUFB8
HIF1AN
PAX2
FAM178A
SEMA4G
MIR608
MRPL43
C10orf2
LZTS2
PDZD7
SFXN3
KAZALD1
TLX1NB
TLX1
LBX1
LBX1-AS1
LOC101927396
LOC101927419
Position on chr10 (Mb)
rs11067773
MED13L
MIR620
Position on chr12 (Mb)
rs112630705
SOX5
MIR920
LOC101928471
LINC00477
BCAT1
C12orf77
LRMP
CASC1
Position on chr12 (Mb)
rs2218650
SYT10
ALG10
Position on chr12 (Mb)
rs4533105
ALG10B
Position on chr12 (Mb)
rs150330648
RGS6
MIR7843
DPF3
Position on chr14 (Mb)
rs12906962
MCTP2
LOC440311
Position on chr15 (Mb)
rs12974991
ZNRF4
TINCR
C19orf70
PRR22
NDUFA11
RFX2
MLLT1
CLPP
SAFB2
LONP1
NRTN
CAPS
ACSBG2
ACER1
SAFB
DUS3L
VMAC
ALKBH7
HSD11B1L
FUT6
RANBP3
PSPN
RPL36
FUT3
LOC100128568
GTF2F1
CATSPERD
FUT5
MIR6885
LOC101928844
MIR6790
Position on chr19 (Mb)

Supplementary Figure 2b: LocusZoom plots for the novel loci for HR response to recovery, $\Delta\mathrm{HR}^{\mathrm{rec}}$, in the full cohort data.

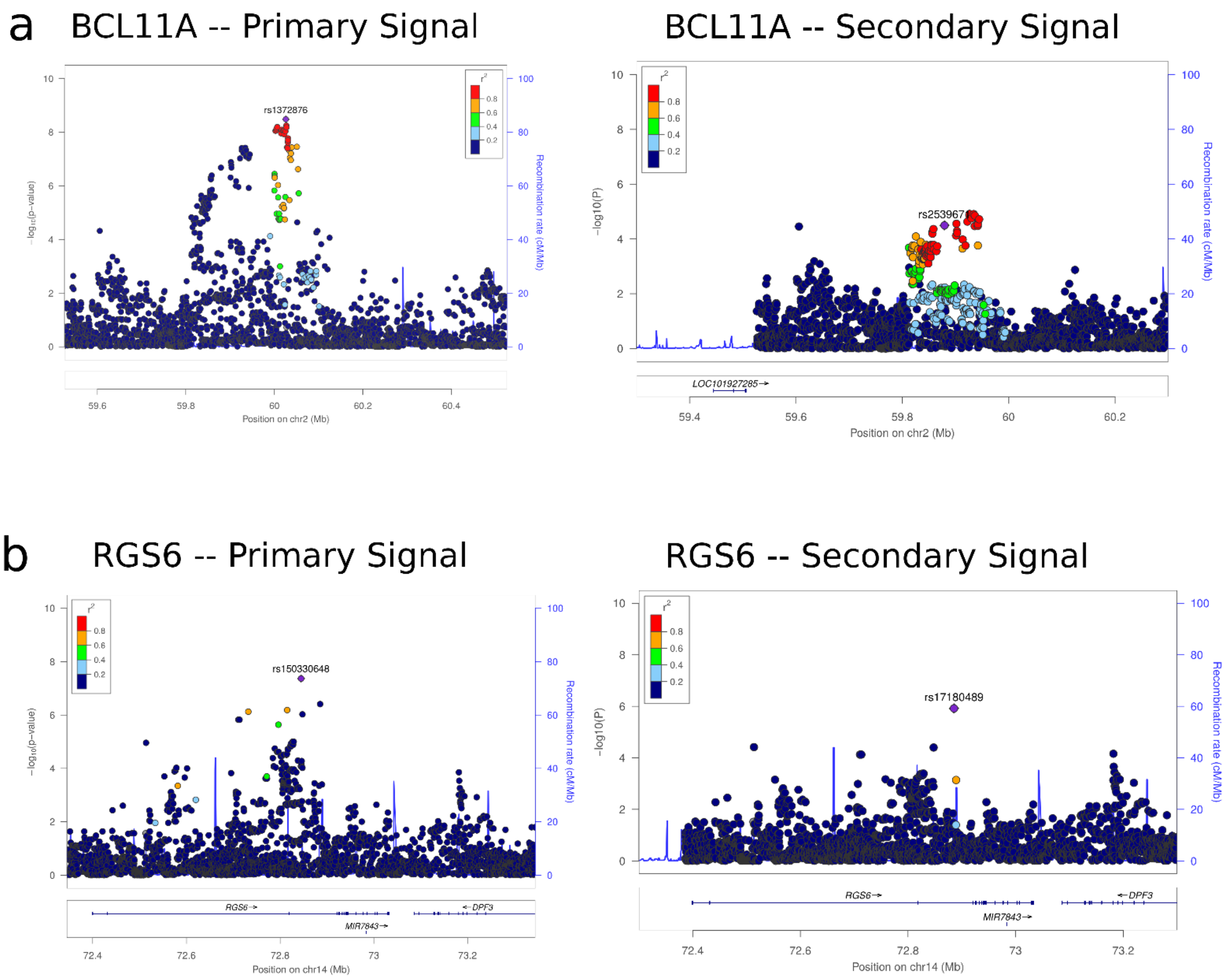

Supplementary Figure 3: Secondary signals at the *BCL11A* (a) and *RGS6* (b) loci contributing to HR response to recovery, $\Delta\mathrm{HR}^{\mathrm{rec}}$.

Left panels show the association plot and linkage disequilibrium (LD) calculations with respect to the primary signal. Right panels show the association plot and LD calculations with respect to the secondary signal after conditional analysis with GCTA. Association plots were calculated using results from the full cohort analysis.

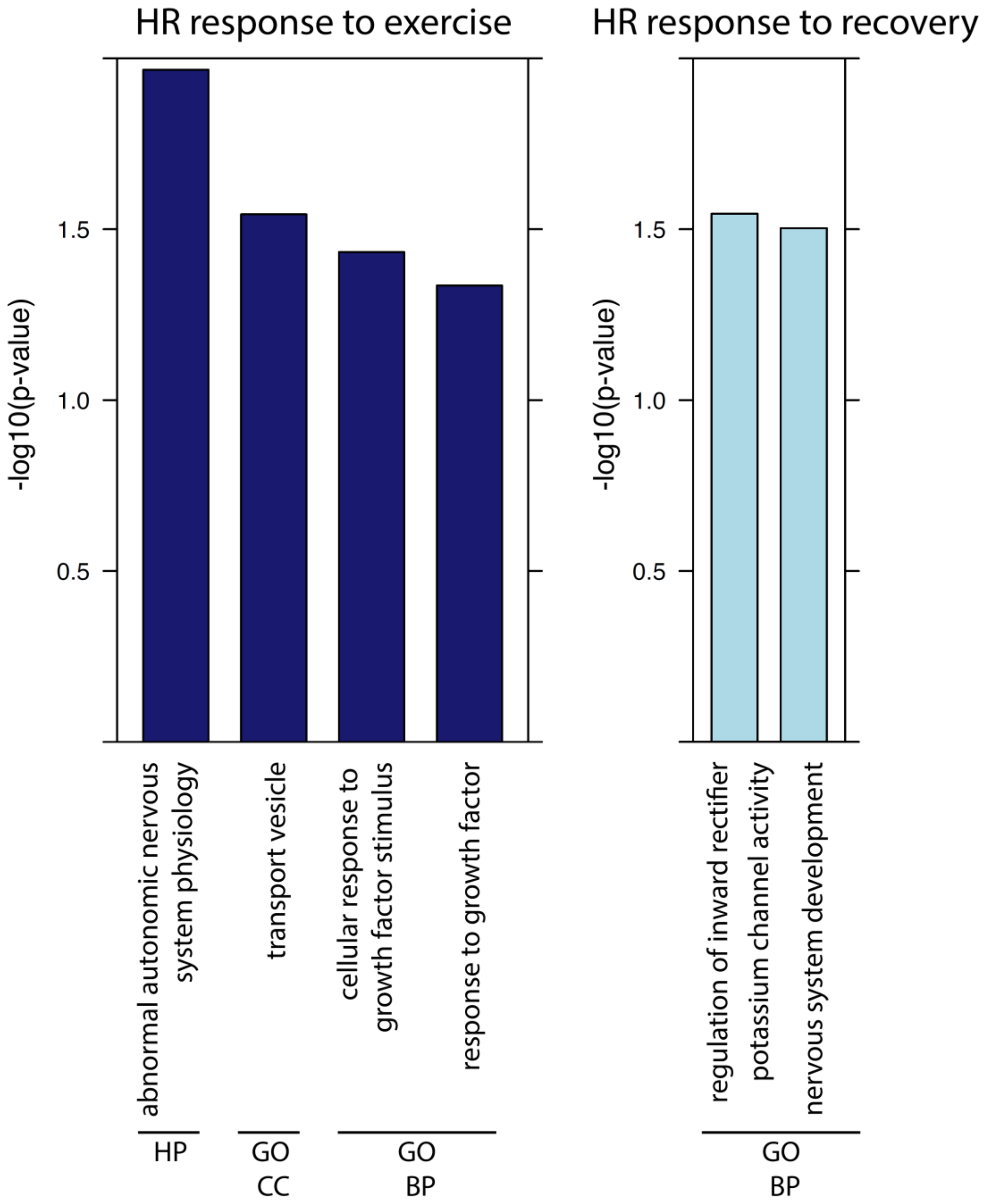

Supplementary Figure 4: Gene list enrichment of candidate genes at HR response to exercise and recovery loci.

gprofiler GO (gene ontology) and HPO (human phenotype ontology) term enrichments were performed using the candidate genes (prioritising genes with known association with heart rate biology, eQTL and long-range target genes). BP- biological pathway, CC-cellular component.

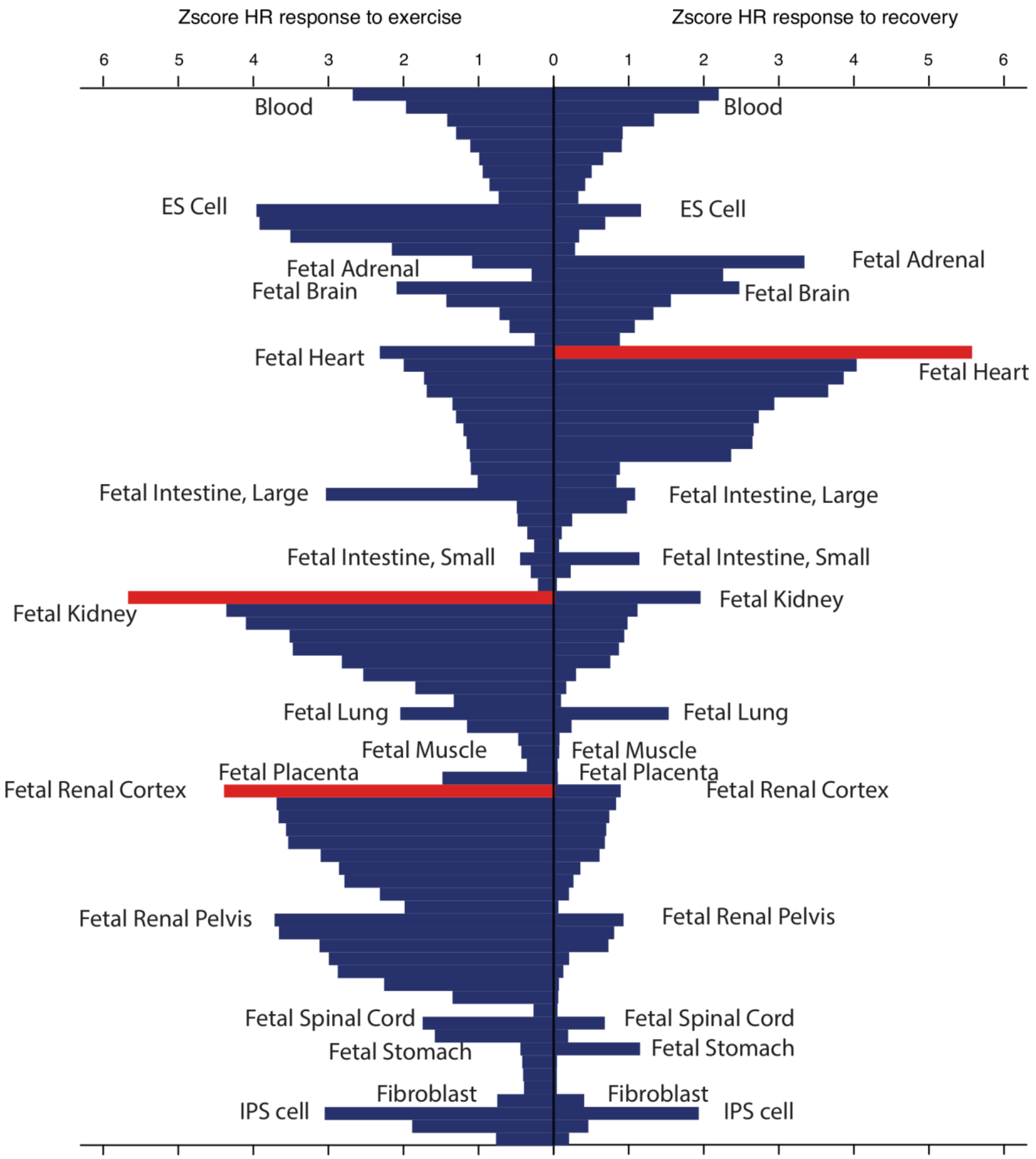

Supplementary Figure 5: Tissue specific enrichment of HR response SNPs in DNase I hypersensitivity sites.

Enrichment across 299 tissue samples from the RoadMap project were assessed. The figure shows samples where the enrichment was above 0 for both HR response to exercise and recovery traits. Samples are sorted for both traits by Z-score/per tissue. Significant enrichments are indicated in red (Bonferroni corrected *P* value < 0.05).

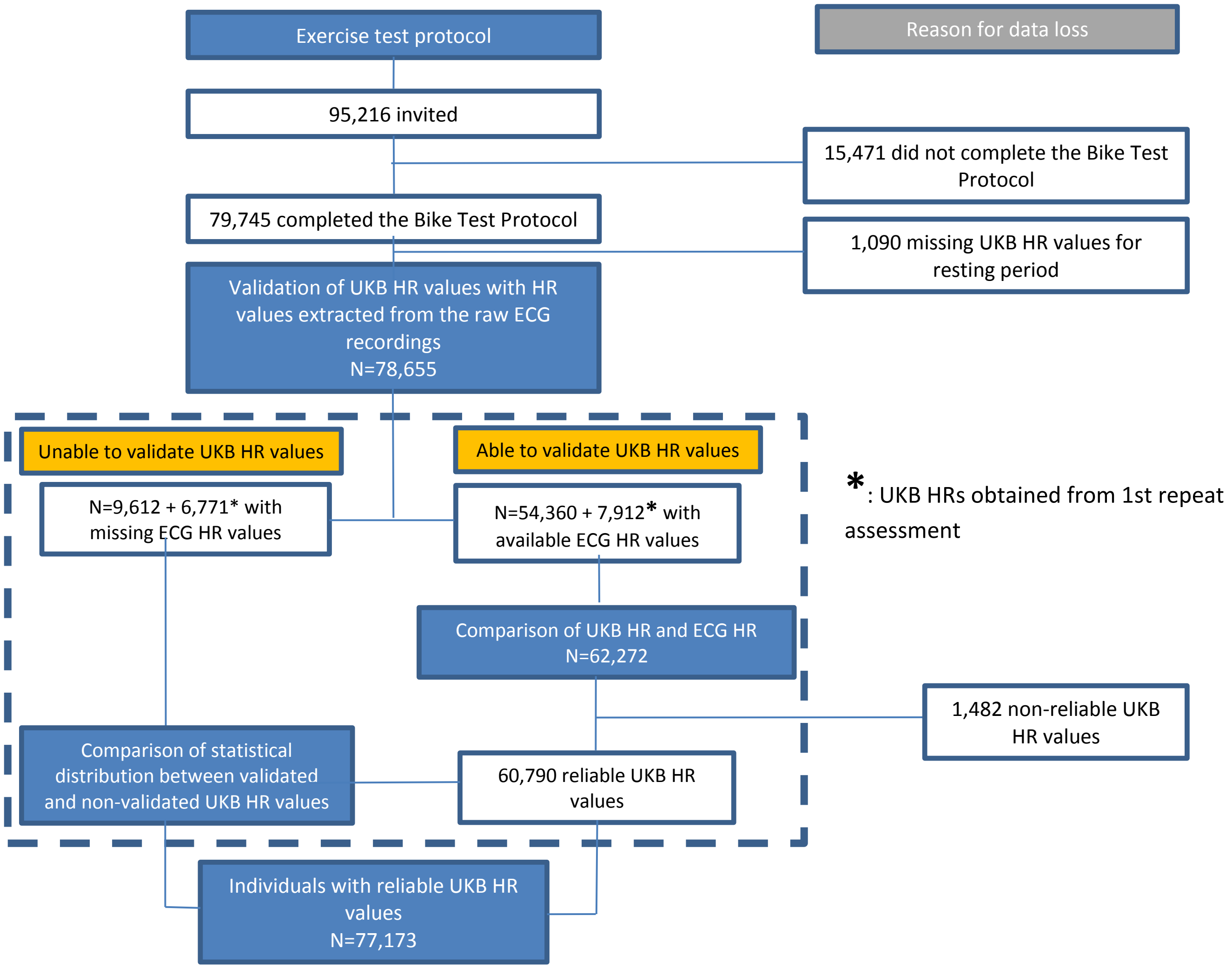

Supplementary Figure 6: Overview of the selection process of individuals who participated in the exercise test.

UKB HR values, i.e. automatic HR values provided by UK Biobank, were obtained from either the initial assessment or, if data was missing, from the first repeat assessment. Data was validated by inspection of ECG HR values, i.e. automatic HR values derived from us by analysis of the raw ECG recordings, if available. All individuals with validated HR data were selected for further genetic analysis.

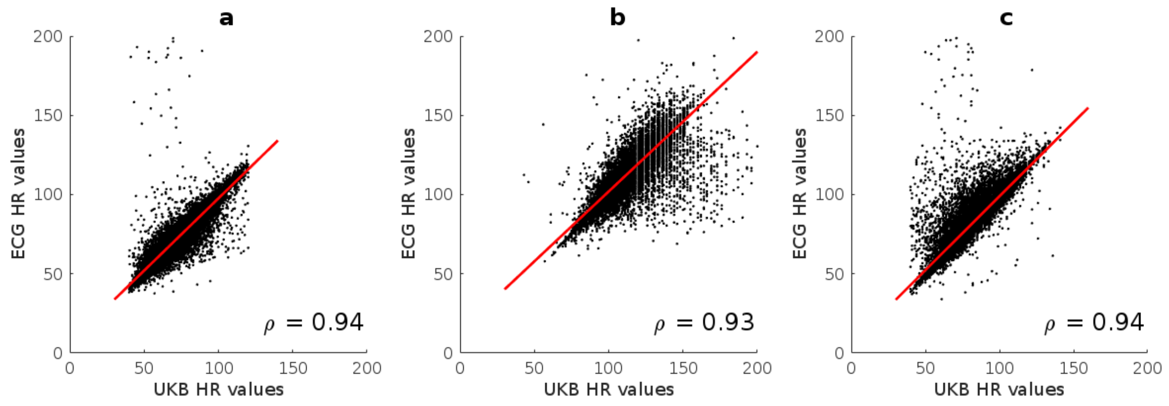

Supplementary Figure 7: Correlation of UKB HR measurements with algorithmically derived HR measurements from raw ECG recordings.

Scatter plots between UKB HR (horizontal axes) and ECG HR (vertical axes) at the three stages of the exercise test, resting (**a**), peak exercise (**b**), and 1 min post-exercise (**c**). The red line shows the corresponding regression line. High Spearman correlation coefficients (ρ) indicate high reliability of the HR showcase method to measure changes in HR.

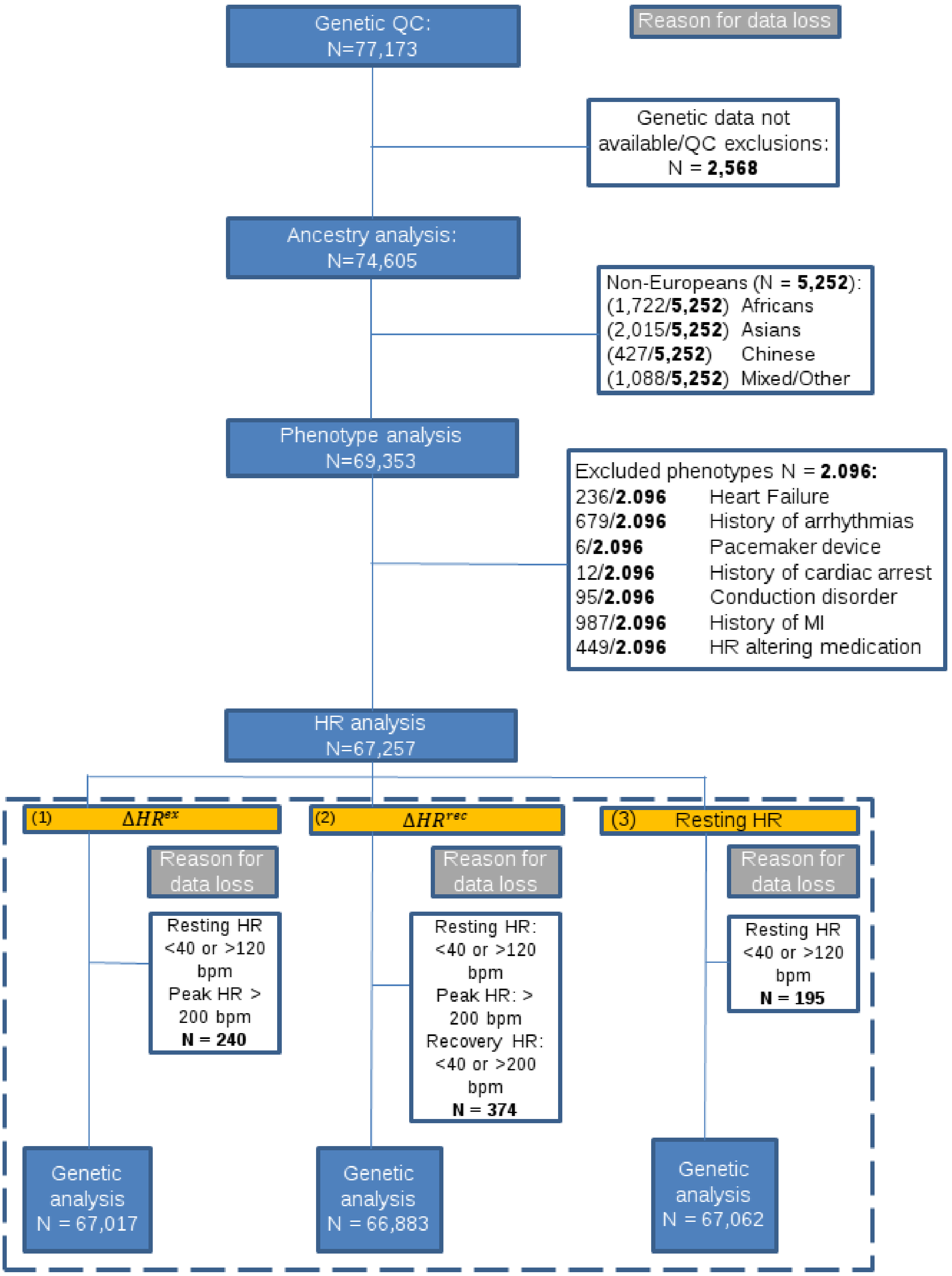

Supplementary Figure 8: Genetic and phenotypic QC for three phenotypic traits, HR response to exercise, ΔHR^{ex}, (1), HR response to recovery, ΔHR^{rec}, (2) and resting HR (3).

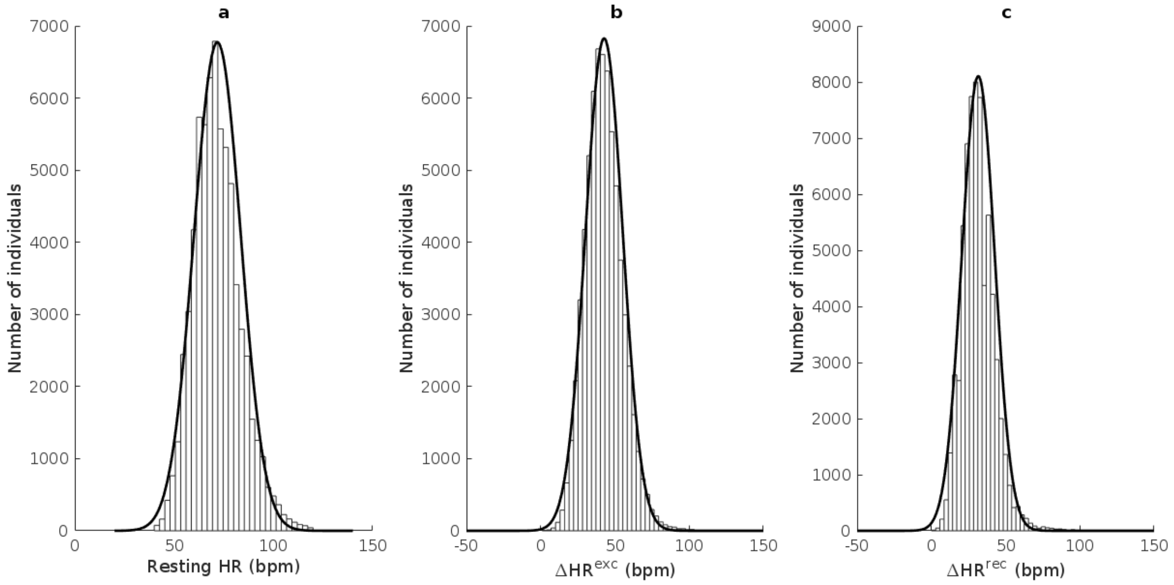

Supplementary Figure 9: Histograms of resting HR (a), HR response to exercise, ΔHR^{ex}, (b) and HR response to recovery, ΔHR^{rec}, (c) after QC.

	Discovery (N=40,000)	Replication (N=26,439)	Full (N=67,257)	*P*
**Age [years]**	57.48 (8.09)	57.52 (8.05)	57.48 (8.09)	0.61
**Males [N (%)]**	18,920 (47.30%)	12,519 (47.35%)	31,808 (47.28%)	0.89
**BMI [kg/m2]**	26.95 (4.37)	26.98 (4.39)	26.96 (4.38)	0.46
**UKB genetic array [N (%)]**	3,800 (9.53%)	2,557 (9.67%)	6,456 (9.60%)	0.53
**Resting HR [bpm]**	71.91 (12.13)	71.86 (12.22)	71.89 (12.15)	0.42
**HR response to exercise [bpm]**	42.76 (12.54)	42.68 (12.60)	42.73 (12.56)	0.25
**HR response to recovery [bpm]**	31.62 (11.08)	31.56 (11.08)	31.60 (11.07)	0.44

Supplementary Table 1: Demographic descriptions of the discovery, replication and full datasets.
Differences between discovery and replication groups were analysed using the Wilcoxon ranked test. Continuous variables are represented as mean (standard deviation). Dichotomised variables are represented as number (percentage). A total of N = 818 individuals were removed from the replication group because they were genetically related (first or second degree, kinship coefficient > 0.88) to individuals from the discovery group. Abbreviations: N: number of participants, P: P-value, BMI: body mass index, HR: heart rate, bpm: beats per minute, UKB: UK Biobank.

Chr	Pos	SNP	EA	AA	EAF	β	SE	*P*	N
5	121861856	rs1478374	T	C	0.818	0.614	0.102	1.70E-09	39322
5	121862860	rs12518676	C	T	0.818	0.618	0.102	1.30E-09	39334
5	121862974	rs12518744	G	T	0.818	0.616	0.102	1.60E-09	39386
5	121863307	rs12519327	C	G	0.818	0.614	0.102	1.70E-09	39354
5	121865362	rs17149297	A	C	0.818	0.615	0.102	1.60E-09	39328
5	121866990	rs4836027	T	C	0.693	0.617	0.086	5.70E-13	39214
5	121868933	rs6595376	A	T	0.693	0.616	0.086	5.90E-13	39220
5	121869310	rs1993875	G	C	0.697	0.602	0.086	2.30E-12	39269
5	121870595	rs11241648	G	A	0.698	0.603	0.086	2.20E-12	39269
5	121871938	rs10519707	G	A	0.693	0.609	0.086	1.10E-12	39267
5	121874008	rs12659801	C	T	0.698	0.593	0.086	4.80E-12	39359
5	121874184	rs12653716	T	C	0.698	0.590	0.086	6.30E-12	39362
5	121874421	rs56051851	C	T	0.787	0.563	0.096	5.50E-09	39382
5	121874730	rs67854110	G	A	0.787	0.564	0.096	4.80E-09	39310
5	121875300	rs4569865	T	C	0.490	-0.436	0.079	3.20E-08	39385
5	121876491	rs11747827	G	A	0.491	-0.436	0.079	3.20E-08	39407
5	121877474	rs55884637	T	C	0.803	0.554	0.099	2.00E-08	39493
5	121883343	rs10037762	G	T	0.490	-0.432	0.079	4.30E-08	39434
5	121900973	rs1122432	T	C	0.808	0.546	0.100	4.60E-08	39558
5	121904581	rs12657010	G	C	0.808	0.547	0.100	4.20E-08	39535
5	121904720	rs17149362	G	A	0.809	0.546	0.100	4.70E-08	39593
5	121905935	rs17149370	G	A	0.809	0.548	0.100	4.40E-08	39584
5	121907799	rs17149379	A	C	0.808	0.553	0.100	3.10E-08	39566
6	122386599	rs2816109	C	T	0.449	-0.475	0.079	1.90E-09	39328
6	122387521	rs9372676	C	T	0.480	-0.444	0.079	1.80E-08	39403
12	24758480	rs4963772	G	A	0.851	-0.694	0.111	3.30E-10	39579
12	24760456	rs34219605	A	G	0.851	-0.694	0.110	3.30E-10	39630
12	24762501	rs11047527	A	C	0.851	-0.693	0.110	3.50E-10	39659

12	24770878	rs17287293	A	G	0.851	-0.698	0.111	2.80E-10	39735
12	24771967	rs10842383	C	T	0.851	-0.700	0.111	2.50E-10	39695
12	24773562	rs12829213	G	A	0.851	-0.701	0.111	2.40E-10	39708
12	24773919	rs112630705	G	A	0.851	-0.701	0.111	2.40E-10	39710
12	24776752	rs137913153	A	G	0.851	-0.701	0.111	2.40E-10	39704
12	24781446	rs11047539	A	G	0.851	-0.701	0.111	2.40E-10	39715
12	24784139	rs4246224	G	A	0.851	-0.702	0.111	2.30E-10	39716
12	24788339	rs11047543	G	A	0.851	-0.684	0.111	5.90E-10	39735
15	95312071	rs12906962	T	C	0.680	0.496	0.085	5.60E-09	39222

Supplementary Table 2: Genome-wide significant SNPs for HR response to exercise in the discovery sample
Abbreviations: SNP: single-nucleotide polymorphism, Chr: Pos: Chromosome: Position, based on HG build 18, EA: Effect allele, AA: Alternate allele, EAF: Effect allele frequency, INFO: imputation information score, β: Beta in beats per minute, se: Standard Error, N: number of participants, *P*: P-value.

						Discovery					Replication					Full			
**Locus**	**SNP**	**CHR**	**BP**	**EA**	**AA**	**EAF**	**β**	**SE**	***P***	**N**	**EAF**	**β**	**SE**	***P***	**N**	**β**	**SE**	***P***	**N**
RNF220	rs272564	1	45012273	A	C	0.716	0.360	0.088	4.30E-05	38757	0.718	0.619	0.108	1.10E-08	25614	0.462	0.068	7.40E-12	65166
CCDC141	rs10497529	2	179839888	G	A	0.964	-1.049	0.212	7.80E-07	39735	0.964	-0.860	0.259	9.10E-04	26261	-0.968	0.162	2.50E-09	66811
SCN10A	rs7433723	3	38784957	G	A	0.420	-0.364	0.080	4.80E-06	39674	0.415	-0.333	0.099	7.40E-04	26221	-0.335	0.061	4.50E-08	66709
SNCA1P	rs4836027	5	121866990	T	C	0.693	0.617	0.086	5.70E-13	39214	0.694	0.599	0.105	1.20E-08	25917	0.613	0.066	9.90E-21	65935
PPIL1	rs236352	6	36817113	A	G	0.341	0.383	0.083	4.20E-06	39213	0.340	0.401	0.103	9.40E-05	25916	0.395	0.064	6.40E-10	65933
CAV2	rs28495552	7	116113744	C	G	0.485	-0.355	0.079	6.40E-06	39671	0.482	-0.467	0.097	1.70E-06	26219	-0.403	0.061	2.80E-11	66703
RP1L1	rs58065122	8	10526186	G	A	0.582	-0.434	0.080	6.60E-08	39187	0.581	-0.294	0.099	2.90E-03	25899	-0.385	0.062	3.90E-10	65889
PAX2	rs11190709	10	102552663	G	A	0.112	0.484	0.125	1.10E-04	39359	0.113	0.852	0.153	2.70E-08	26013	0.649	0.096	1.30E-11	66180
LINC00477	rs4246224	12	24784139	G	A	0.851	-0.702	0.111	2.30E-10	39716	0.849	-0.615	0.135	5.40E-06	26248	-0.649	0.085	1.80E-14	66778
SYT10	rs1343676	12	33537387	T	C	0.494	-0.408	0.079	2.20E-07	39537	0.494	-0.404	0.097	3.10E-05	26130	-0.407	0.060	1.50E-11	66477
HMGA2	rs1480470	12	66412130	A	G	0.371	0.367	0.082	7.90E-06	39139	0.373	0.294	0.100	3.30E-03	25867	0.347	0.063	3.40E-08	65810
MCTP2	rs12906962	15	95312071	T	C	0.680	0.496	0.085	5.60E-09	39222	0.679	0.461	0.105	1.10E-05	25922	0.475	0.065	3.50E-13	65949

TCF4	rs1125313	18	52859261	A	C	0.501	-0.349	0.079	1.10E-05	39348	0.500	-0.375	0.098	1.30E-04	26005	-0.359	0.061	3.90E-09	66161
POP4	rs7255293	19	30104198	A	G	0.580	0.354	0.080	9.60E-06	39405	0.579	0.352	0.098	3.40E-04	26043	0.363	0.061	3.20E-09	66257

Supplementary Table 3: Discovery, replication, and full GWAS results for all reported SNPs for HR response to exercise
Abbreviations: SNP: single-nucleotide polymorphism, CHR: Chromosome, BP: Base pair Position, based on HG build 18, EA: Effect allele, AA: Alternate allele, EAF: Effect allele frequency, β: Beta in beats per minute, SE: Standard Error, N: effective number of participants, *P*: P-value. The locus name indicates the gene that is in the closest proximity to the most associated SNP.

						**Discovery**					**Replication**					**Full**			
**Locus**	**SNP**	**CHR**	**BP**	**EA**	**AA**	**EAF**	**β**	**SE**	***P***	**N**	**EAF**	**β**	**SE**	***P***	**N**	**β**	**SE**	***P***	**N**
RNF220	rs272564	1	45012273	A	C	0.716	0.359	0.079	5.20E-06	38674	0.718	0.391	0.096	5.00E-05	25571	0.370	0.060	8.80E-10	65036
BCL11A	rs1372876	2	60025963	A	C	0.414	-0.350	0.072	1.10E-06	38731	0.412	-0.271	0.088	2.10E-03	25608	-0.326	0.055	3.30E-09	65133
SCN10A	rs6795970	3	38766675	A	G	0.404	-0.360	0.072	4.80E-07	39650	0.399	-0.244	0.088	5.60E-03	26216	-0.306	0.055	2.60E-08	66678
CNTN3	rs6549649	3	74786491	G	C	0.559	-0.375	0.071	1.20E-07	39438	0.557	-0.275	0.087	1.50E-03	26076	-0.328	0.054	1.40E-09	66321
SNCA1P	rs1993875	5	121869310	G	C	0.697	0.339	0.077	1.00E-05	39185	0.698	0.352	0.094	1.80E-04	25908	0.338	0.059	9.50E-09	65895
ACHE	rs3757868	7	100482720	G	A	0.815	0.396	0.090	1.10E-05	39602	0.820	0.539	0.112	1.50E-06	26184	0.454	0.070	6.90E-11	66597
CAV2	rs2109514	7	116159961	G	A	0.501	-0.391	0.071	3.10E-08	39462	0.499	-0.259	0.087	2.80E-03	26092	-0.334	0.054	7.10E-10	66362
CHRM2	rs6943656	7	136639436	A	G	0.842	0.466	0.097	1.40E-06	39113	0.840	0.462	0.119	1.00E-04	25861	0.470	0.074	2.30E-10	65775
PAX2	rs4917911	10	102559421	G	A	0.111	0.622	0.112	2.60E-08	39470	0.113	0.673	0.136	7.10E-07	26097	0.665	0.085	6.60E-15	66376
LINC00477-SOX5	rs112630705	12	24773919	G	A	0.851	-0.565	0.099	1.10E-08	39625	0.849	-0.397	0.120	9.70E-04	26199	-0.502	0.076	3.20E-11	66636
SYT10	rs2218650	12	33734783	A	G	0.641	0.642	0.073	2.50E-18	39522	0.640	0.545	0.090	1.40E-09	26131	0.602	0.056	1.10E-26	66463
ALG10B	rs4533105	12	38214611	T	C	0.570	0.411	0.072	1.00E-08	38916	0.571	0.392	0.088	8.20E-06	25731	0.404	0.055	1.90E-13	65444
MED13L	rs11067773	12	116228495	T	C	0.910	0.621	0.123	4.70E-07	39403	0.911	0.635	0.152	2.80E-05	26053	0.628	0.095	3.10E-11	66262

RGS6	rs150330648	14	72844765	G	T	0.987	1.734	0.331	1.70E-07	35768	0.988	0.765	0.408	6.10E-02	23649	1.395	0.255	4.30E-08	60150
MCTP2	rs12906962	15	95312071	T	C	0.680	0.425	0.076	2.40E-08	39139	0.679	0.218	0.093	1.90E-02	25878	0.341	0.058	5.10E-09	65818
NDUFA11	rs12974991	19	5894584	G	A	0.912	0.512	0.123	3.30E-05	39532	0.911	0.616	0.152	5.20E-05	26138	0.568	0.095	2.10E-09	66479

Supplementary Table 4: Discovery, replication, and full GWAS results for all reported SNPs for HR response to recovery
Abbreviations: SNP: single-nucleotide polymorphism, CHR: Chromosome, BP: Base pair Position, based on HG build 18, EA: Effect allele, AA: Alternate allele, EAF: Effect allele frequency, β: Beta in beats per minute, SE: Standard Error, N: effective number of participants, *P*: P-value. The locus name indicates the gene that is in the closest proximity to the most associated SNP.

A. HR response to exercise						Including individuals taking beta blockers				Excluding individuals taking beta blockers			
Locus	SNP	CHR	BP	EA	AA	β	SE	*P*	N	β	SE	*P*	N
RNF220	rs272564	1	45012273	A	C	0.462	0.068	7.40E-12	65166	0.458	0.068	2.00E-11	62608
CCDC141	rs10497529	2	179839888	G	A	-0.968	0.162	2.50E-09	66811	-1.027	0.164	4.00E-10	64188
SCN10A	rs7433723	3	38784957	G	A	-0.335	0.061	4.50E-08	66709	-0.344	0.062	2.80E-08	64090
SNCA1P	rs4836027	5	121866990	T	C	0.613	0.066	9.90E-21	65935	0.597	0.066	2.40E-19	63346
PPIL1	rs236352	6	36817113	A	G	0.395	0.064	6.40E-10	65933	0.394	0.065	1.10E-09	63345
CAV2	rs28495552	7	116113744	C	G	-0.403	0.061	2.80E-11	66703	-0.397	0.061	9.00E-11	64084
RP1L1	rs58065122	8	10526186	G	A	-0.385	0.062	3.90E-10	65889	-0.378	0.062	1.30E-09	63303
PAX2	rs11190709	10	102552663	G	A	0.649	0.096	1.30E-11	66180	0.650	0.097	2.20E-11	63581
LINC00477	rs4246224	12	24784139	G	A	-0.649	0.085	1.80E-14	66778	-0.610	0.086	1.10E-12	64157
SYT10	rs1343676	12	33537387	T	C	-0.407	0.060	1.50E-11	66477	-0.403	0.061	4.30E-11	63867
HMGA2	rs1480470	12	66412130	A	G	0.347	0.063	3.40E-08	65810	0.338	0.064	1.10E-07	63226
MCTP2	rs12906962	15	95312071	T	C	0.475	0.065	3.50E-13	65949	0.453	0.066	8.00E-12	63360
TCF4	rs1125313	18	52859261	A	C	-0.359	0.061	3.90E-09	66161	-0.381	0.062	7.00E-10	63563
POP4	rs7255293	19	30104198	A	G	0.363	0.061	3.20E-09	66257	0.334	0.062	7.80E-08	63656
**B. HR response to recovery**						**Including individuals taking beta blockers**				**Excluding individuals taking beta blockers**			
**Locus**	**SNP**	**CHR**	**BP**	**EA**	**AA**	**β**	**SE**	***P***	**N**	**β**	**SE**	***P***	**N**
RNF220	rs272564	1	45012273	A	C	0.370	0.060	8.80E-10	65036	0.364	0.061	3.00E-09	62480
BCL11A	rs1372876	2	60025963	A	C	-0.326	0.055	3.30E-09	65133	-0.294	0.056	1.60E-07	62572
SCN10A	rs6795970	3	38766675	A	G	-0.306	0.055	2.60E-08	66678	-0.328	0.056	4.10E-09	64057
CNTN3	rs6549649	3	74786491	G	C	-0.328	0.054	1.40E-09	66321	-0.336	0.055	1.10E-09	63714
SNCA1P	rs1993875	5	121869310	G	C	0.338	0.059	9.50E-09	65895	0.326	0.060	4.70E-08	63305
ACHE	rs3757868	7	100482720	G	A	0.454	0.070	6.90E-11	66597	0.458	0.071	9.30E-11	63979
CAV2	rs2109514	7	116159961	G	A	-0.334	0.054	7.10E-10	66362	-0.326	0.055	3.10E-09	63754

CHRM2	rs6943656	7	136639436	A	G	0.470	0.074	2.30E-10	65775	0.343	0.063	5.50E-08	63193
PAX2	rs4917911	10	102559421	G	A	0.665	0.085	6.60E-15	66376	0.682	0.087	3.80E-15	63767
LINC00477-SOX5	rs112630705	12	24773919	G	A	-0.502	0.076	3.20E-11	66636	-0.463	0.077	1.60E-09	64016
SYT10	rs2218650	12	33734783	A	G	0.602	0.056	1.10E-26	66463	0.585	0.057	1.70E-24	63850
ALG10B	rs4533105	12	38214611	T	C	0.404	0.055	1.90E-13	65444	0.392	0.056	2.30E-12	62871
MED13L	rs11067773	12	116228495	T	C	0.628	0.095	3.10E-11	66262	0.646	0.096	1.70E-11	63658
RGS6	rs150330648	14	72844765	G	T	1.395	0.255	4.30E-08	60150	1.357	0.259	1.50E-07	57785
MCTP2	rs12906962	15	95312071	T	C	0.341	0.058	5.10E-09	65818	0.344	0.059	6.80E-09	63231
NDUFA11	rs12974991	19	5894584	G	A	0.568	0.095	2.10E-09	66479	0.573	0.097	2.90E-09	63866

Supplementary Table 5: Comparison of results for all loci associated with HR response to exercise and to recovery traits in individuals not taking beta-blockers.

Abbreviations: SNP: single-nucleotide polymorphism, CHR: Chromosome, BP: Base pair Position, based on HG build 18, EA: Effect allele, AA: Alternate allele, EAF: Effect allele frequency, β: Beta in beats per minute, SE: Standard Error, N: effective number of participants, P: P-value. The locus name indicates the gene that is in the closest proximity to the most associated SNP.

**A. HR response to exercise**												
							**Full GWAS analyses**					
**Locus**	**SNP**	**CHR**	**BP**	**EA**	**AA**	**EAF**	**β**	**SE**	***P***	**N**	***P* for resting HR**	**N**
RNF220*	rs272564	1	45012273	A	C	0.717	0.462	0.068	7.40E-12	65166	1.40E-07	65397
CCDC141	rs10497529	2	179839888	G	A	0.964	-0.968	0.162	2.50E-09	66811	5.30E-15	67048
SCN10A~	rs7433723	3	38784957	G	A	0.418	-0.335	0.061	4.50E-08	66709	1.20E-05	66946
SNCA1P~	rs4836027	5	121866990	T	C	0.693	0.613	0.066	9.90E-21	65935	3.60E-01	66169
PPIL1	rs236352	6	36817113	A	G	0.340	0.395	0.064	6.40E-10	65933	4.40E-02	66167
CAV2~	rs28495552	7	116113744	C	G	0.484	-0.403	0.061	2.80E-11	66703	6.30E-01	66940
RP1L1	rs58065122	8	10526186	G	A	0.582	-0.385	0.062	3.90E-10	65889	1.90E-01	66123
PAX2~	rs11190709	10	102552663	G	A	0.112	0.649	0.096	1.30E-11	66180	1.20E-02	66414
LINC00477-SOX5~	rs4246224	12	24784139	G	A	0.851	-0.649	0.085	1.80E-14	66778	2.40E-17	67015
SYT10^	rs1343676	12	33537387	T	C	0.494	-0.407	0.060	1.50E-11	66477	3.60E-12	66713
HMGA2	rs1480470	12	66412130	A	G	0.372	0.347	0.063	3.40E-08	65810	6.50E-02	66043
MCTP2*	rs12906962	15	95312071	T	C	0.680	0.475	0.065	3.50E-13	65949	1.90E-02	66183
TCF4	rs1125313	18	52859261	A	C	0.500	-0.359	0.061	3.90E-09	66161	3.40E-02	66395
POP4	rs7255293	19	30104198	A	G	0.579	0.363	0.061	3.20E-09	66257	8.70E-01	66492
**B. HR response to recovery**												
							**Full GWAS analyses**					
**Locus**	**SNP**	**CHR**	**BP**	**EA**	**AA**	**EAF**	**β**	**SE**	**P**	**N**	**P for resting HR**	**N**
RNF220*	rs272564	1	45012273	A	C	0.717	0.370	0.060	8.80E-10	65036	1.40E-07	65397
BCL11A	rs1372876	2	60025963	A	C	0.413	-0.326	0.055	3.30E-09	65133	8.60E-02	65494
SCN10A~	rs6795970	3	38766675	A	G	0.402	-0.306	0.055	2.60E-08	66678	3.90E-05	67048
CNTN3	rs6549649	3	74786491	G	C	0.558	-0.328	0.054	1.40E-09	66321	8.60E-01	66689
SNCA1P~	rs1993875	5	121869310	G	C	0.698	0.338	0.059	9.50E-09	65895	4.60E-01	66261
ACHE	rs3757868	7	100482720	G	A	0.817	0.454	0.070	6.90E-11	66597	8.10E-14	66966
CAV2~	rs2109514	7	116159961	G	A	0.500	-0.334	0.054	7.10E-10	66362	8.60E-01	66731

CHRM2	rs6943656	7	136639436	A	G	0.842	0.470	0.074	2.30E-10	65775	9.30E-09	67048
PAX2~	rs4917911	10	102559421	G	A	0.112	0.665	0.085	6.60E-15	66376	1.40E-02	66744
LINC00477-SOX5~	rs112630705	12	24773919	G	A	0.851	-0.502	0.076	3.20E-11	66636	2.20E-17	67005
SYT10^	rs2218650	12	33734783	A	G	0.641	0.602	0.056	1.10E-26	66463	8.10E-11	66832
ALG10B	rs4533105	12	38214611	T	C	0.570	0.404	0.055	1.90E-13	65444	7.40E-05	65807
MED13L	rs11067773	12	116228495	T	C	0.911	0.628	0.095	3.10E-11	66262	6.90E-01	66630
RGS6	rs150330648	14	72844765	G	T	0.988	1.395	0.255	4.30E-08	60150	2.30E-02	60483
MCTP2*	rs12906962	15	95312071	T	C	0.680	0.341	0.058	5.10E-09	65818	1.90E-02	66183
NDUFA11	rs12974991	19	5894584	G	A	0.912	0.568	0.095	2.10E-09	66479	5.60E-03	66848

Supplementary Table 6: Association results of SNPs associated with (A) HR response to exercise (B) HR response to recovery in resting HR GWAS.

Abbreviations: SNP: single-nucleotide polymorphism, CHR: Chromosome, BP: Base pair Position, based on HG build 18, EA: Effect allele, AA: Alternate allele, EAF: Effect allele frequency, β: Beta in beats per minute, SE: Standard Error, N: effective number of participants, P: P-value.
The locus name indicates the gene that is in the closest proximity to the most associated SNP.
* indicates if it is the same SNP across HR response traits.
~ indicates if SNPs between HR response to exercise and HR response to recovery traits are in high LD (r2 >0. 80).
^ indicates if SNPs between HR response to exercise and HR response to recovery traits are in low LD (0.8 > r2 > 0.3)

Bold rows indicate genome-wide significant SNP for resting HR in the full dataset GWAS.

A. HR response to exercise							
**Locus**	**SNP**	**CHR**	**BP**	**EA**	**EAF**	**LD (r^2) with published resting HR or HRV SNV**	**Distance**
**RNF220**	**rs272564**	**1**	**45012273**	**A**	**0.284**	**1.00**	**Within a 500 KB distance from a primary resting HR SNP**
CCDC141	rs10497529	2	179839888	A	0.976	< 0.1	Within a 500 KB distance from a secondary resting HR SNP
SCN10A	rs7433723	3	38784957	A	0.418	< 0.1	Within a 500 KB distance from a secondary resting HR SNP
SNCA1P	rs4836027	5	121866990	T	0.693	< 0.1	More than 500 KB apart from any primary or secondary resting HR or HRV SNP
**PPIL1**	**rs236352**	**6**	**36817113**	**A**	**0.340**	**1.00**	**Within a 500 KB distance from a primary resting HR and HRV SNP**
CAV2	rs28495552	7	116113744	C	0.484	< 0.1	Within a 500 KB distance from a primary resting HR SNP
RP1L1	rs58065122	8	10526186	A	0.487	< 0.1	More than 500 KB appart from any primary or secondary resting HR or HRV SNP
PAX2	rs11190709	10	102552663	A	0.112	< 0.1	Within a 500 KB distance from a primary resting HR SNP
**LINC00477**	**rs4246224**	**12**	**24784139**	**A**	**0.851**	**0.99**	**Within a 500 KB distance from a primary resting HR SNP**
SYT10	rs1343676	12	33537387	T	0.494	0.62	Within a 500 KB distance from a primary resting HR SNP
HMGA2	rs1480470	12	66412130	A	0.372	< 0.1	More than 500 KB apart from any primary or secondary resting HR or HRV SNP
MCTP2	rs12906962	15	95312071	T	0.680	< 0.1	More than 500 KB apart from any primary or secondary resting HR or HRV SNP
TCF4	rs1125313	18	52859261	A	0.500	< 0.1	More than 500 KB apart from any primary or secondary resting HR or HRV SNP
POP4	rs7255293	19	30104198	A	0.579	< 0.1	More than 500 KB apart from any primary or secondary resting HR or HRV SNP

**B. HR response to recovery**							
**Locus**	**SNP**	**CHR**	**BP**	**EA**	**EAF**	**LD (r^2) with published resting HR or HRV SNV**	**Distance**
**RNF220**	**rs272564**	**1**	**45012273**	**A**	**0.284**	**1.00**	**Within a 500 KB distance from a primary resting HR SNP**
**BCL11A**	**rs1372876**	**2**	**60025963**	**A**	**0.414**	**0.83**	**Within a 500 KB distance from a primary resting HR SNP**
SCN10A	rs6795970	3	38766675	A	0.404	< 0.1	Within a 500 KB distance from a primary resting HR SNP
CNTN3	rs6549649*	3	74786491	G	0.441	< 0.1	More than 500 KB apart from any primary or secondary resting HR or HRV SNP
SNCA1P	rs1993875*	5	121869310	G	0.303	< 0.1	More than 500 KB apart from any primary or secondary resting HR or HRV SNP
**ACHE**	**rs3757868**	**7**	**100482720**	**G**	**0.185**	**0.99**	**Within a 500 KB distance from a primary resting HR SNP**
CAV2	rs2109514	7	116159961	G	0.499	< 0.1	Within a 500 KB distance from a primary resting HR SNP
CHRM2	rs6943656	7	136639436	A	0.842	0.72	Within a 500 KB distance from a primary resting HR SNP
PAX2	rs4917911	10	102559421	G	0.111	< 0.1	Within a 500 KB distance from a primary resting HR SNP
**LINC00477**	**rs112630705**	**12**	**24773919**	**G**	**0.149**	**0.99**	**Within a 500 KB distance from a primary resting HR SNP**
SYT10	rs2218650	12	33734783	A	0.359	0.57	Within a 500 KB distance from a primary resting HR SNP
**ALG10B**	**rs4533105**	**12**	**38214611**	**T**	**0.430**	**0.91**	**Within a 500 KB distance from a primary resting HR SNP**
MED13L	rs11067773*	12	116228495	T	0.090	< 0.1	More than 500 KB apart from any primary or secondary resting HR or HRV SNP
RGS6	rs150330648	14	72844765	G	0.987	<0.1	Within a 500 KB distance from a primary resting HR SNP
MCTP2	rs12906962*	15	95312071	T	0.680	< 0.1	More than 500 KB apart from any primary or secondary resting HR or HRV SNP

**NDUFA11**	**rs12974991**	**19**	**5894584**	**G**	**0.088**	**1**	**Within a 500 KB distance from a primary resting HR SNP**

Supplementary Table 7: Genetic correlation of HR response to exercise and to recovery SNPs and resting HR or HRV loci.
Abbreviations: SNP: single-nucleotide polymorphism, CHR: chromosome, BP: Base pair position, based on HG built 18, EA: effect allele, EAF: effect allele frequency, LD: Linkage disequilibrium, KB: Kilobase, HRV: Heart rate variability. The locus name indicates the gene that is in the closest proximity to the most associated SNP.
Bold: SNPs near and in high LD (r2 > 0.8) with published resting HR or HRV SNPs

**A. HR response to exercise**						**MALES**				**FEMALES**			
**SNP**	**CHR**	**BP**	**EA**	**AA**	**EAF**	**β**	**SE**	**P**	**N**	**β**	**SE**	**P**	**N**
**rs272564**	**1**	**45012273**	**A**	**C**	**0.718759**	**0.547538**	**0.0937357**	**5.20E-09**	**30779**	0.397	0.097	4.20E-05	34294
**rs4836027**	**5**	**121866990**	**T**	**C**	**0.693289**	**0.630437**	**0.090511**	**3.30E-12**	**31142**	**0.611**	**0.095**	**1.10E-10**	**34699**
**rs6595376**	**5**	**121868933**	**A**	**T**	**0.693**	**0.627**	**0.090**	**4.30E-12**	**31147**	**0.614**	**0.095**	**9.00E-11**	**34704**
**rs9270779***	**6**	**32569056**	**T**	**C**	**0.323**	0.095	0.091	3.00E-01	30132	**-0.538**	**0.094**	**1.20E-08**	**33573**
**rs28495552**	**7**	**116113744**	**C**	**G**	**0.482**	-0.294	0.084	4.60E-04	31505	**-0.477**	**0.087**	**4.10E-08**	**35103**
**rs11775702**	**8**	**10517347**	**C**	**G**	**0.578**	-0.199	0.085	1.90E-02	31172	**-0.506**	**0.089**	**1.10E-08**	**34733**
**rs60717250***	**8**	**121050233**	**C**	**T**	**0.630651**	**0.485723**	**0.086513**	**2.00E-08**	**31454**	0.026	0.090	7.70E-01	35047
**rs10883543**	**10**	**102552752**	**G**	**T**	**0.111801**	**0.754234**	**0.132641**	**1.30E-08**	**31221**	0.562	0.139	4.90E-05	34787
**rs137913153**	**12**	**24776752**	**A**	**G**	**0.851894**	**-0.782854**	**0.117765**	**3.00E-11**	**31532**	-0.554	0.121	5.00E-06	35133
**rs12906962**	**15**	**95312071**	**T**	**C**	**0.680744**	**0.607292**	**0.0904899**	**1.90E-11**	**31149**	0.353	0.094	1.70E-04	34706
**B. HR response to recovery**													
						**MALES**				**FEMALES**			
**SNP**	**CHR**	**BP**	**EA**	**AA**	**EAF**	**β**	**SE**	**P**	**N**	**β**	**SE**	**P**	**N**
**rs197084**	**2**	**59941979**	**A**	**G**	**0.577**	0.117	0.074	1.10E-01	31092	**0.442**	**0.08**	**3.30E-08**	**34685**
**rs10883543**	**10**	**102552752**	**G**	**T**	**0.112**	**0.688**	**0.116**	**3.00E-09**	**31183**	0.649	0.125	2.20E-07	34787
**rs1351682**	**12**	**33598775**	**G**	**A**	**0.467**	**-0.609**	**0.073**	**7.30E-17**	**31441**	**-0.569**	**0.079**	**5.30E-13**	**35074**
**rs1384590**	**12**	**33734271**	**C**	**T**	**0.642**	**0.498**	**0.076**	**6.20E-11**	**31421**	**0.69**	**0.082**	**4.60E-17**	**35052**
**rs10880321**	**12**	**38370110**	**C**	**G**	**0.379**	-0.335	0.076	1.00E-05	31106	**-0.49**	**0.081**	**1.70E-09**	**34701**

Supplementary Table 8: Sex-stratified analysis for HR response to exercise and HR to recovery traits
Abbreviations: SNP: single-nucleotide polymorphism, CHR: chromosome, BP: Base pair position, based on HG built 18, EA: effect allele, AA: alternate allele, EAF: effect allele frequency, β: Beta in beats per minute, SE: Standard Error, N: effective number of participants, P: P-value.
* Indicates Not in LD with any published resting HR or HRV SNV or with any discovered SNV from our main analyses

Locus	Lead SNP	Proxy SNP	Proxy Chr:Pos (hg19)	EA	r2	Type	Trait	*P*	PMID
RNF220	rs272564	rs156653	chr1:45003255	T/C	0.854	Proxy	Serum concentration of epiandrosterone sulfate	6.30E-05	21886157
BCL11A	rs1372876	rs1372876	chr2:60025963	C/A	1	Lead	Bipolar disorder and schizophrenia	2.25E-05	20451256
	rs1372876	rs12713404	chr2:60006705	T/G	0.84	Proxy	Late onset Alzheimers disease	5.66E-05	20885792
SCN10A	rs7433723	rs6800541	chr3:38774832	T/C	0.952	Proxy	**PR interval**	**9.70E-82**	20062060
	rs7433723	rs6800541	chr3:38774832	T/C	0.952	Proxy	**Electrocardiography**	**2.00E-74**	20062060
	rs7433723	rs10428132	chr3:38777554	G/T	0.972	Proxy	**Brugada syndrome**	**1.00E-68**	23872634
	rs7433723	rs6795970	chr3:38766675	G/A	0.921	Proxy	**Electrocardiographic traits**	**1.00E-58**	20062063
	rs7433723	rs6801957	chr3:38767315	C/T	0.96	Proxy	**PR segment**	**7.00E-41**	24850809
	rs7433723	rs6800541	chr3:38774832	T/C	0.952	Proxy	**QRS interval**	**5.85E-29**	21076409
	rs7433723	rs6801957	chr3:38767315	C/T	0.96	Proxy	**QRS duration**	**1.00E-28**	21076409
	rs7433723	rs6801957	chr3:38767315	C/T	0.96	Proxy	**P wave duration**	**8.00E-27**	24850809
	rs7433723	rs6801957	chr3:38767315	C/T	0.96	Proxy	**Heart Function Tests**	**3.00E-14**	21076409
	rs7433723	rs6800541	chr3:38774832	T/C	0.952	Proxy	Atrioventricular conduction	5.00E-07	21041692
	rs7433723	rs6798015	chr3:38798836	T/C	0.816	Proxy	Nonsyndromic cleft lip	1.33E-05	22863734
	rs7433723	rs6795970	chr3:38766675	G/A	0.921	Proxy	Atrial fibrillation	2.20E-05	22544366
	rs7433723	rs6783110	chr3:38752935	G/A	0.84	Proxy	Serum ratio of hexadecanedioatetaurod eoxycholate	6.20E-05	21886157
PPIL1	rs236352	rs4594944	chr6:36840767	A/G	0.825	Proxy	Heart rate	3.98E-05	23583979
HLA-DRB5/HLA-DRB1	rs9270779	rs9270779	chr6:32569056	C/T	1	Lead	**Inflammatory bowel disease**	**3.20E-22**	26192919
	rs9270779	rs9270779	chr6:32569056	C/T	1	Lead	**Ulcerative colitis**	**3.57E-35**	26192919

	rs9270779	rs9270779	chr6:32569056	C/T	1	Lead	Alzheimers disease	0.0001591	25056061
	rs9270779	rs9270779	chr6:32569056	C/T	1	Lead	Schizophrenia	1.11E-05	24162737
ACHE	rs3757868	rs3757868	chr7:100482720	A/G	1	Lead	**Heart rate**	**2.27E-13**	23583979
	rs3757868	rs12705092	chr7:100473550	A/G	0.993	Proxy	**RR interval**	**2.13E-09**	20639392
	rs3757868	rs12705095	chr7:100484381	G/T	1	Proxy	Total cholesterol	4.30E-05	24097068
CAV2	rs28495552	rs2270189	chr7:116140616	A/G	0.926	Proxy	Primary open angle glaucoma	1.20E-05	20835238
RP1L1	rs58065122	rs11775702	chr8:10517347	G/C	0.96	Proxy	Neuroticism	5.23E-05	27089181
PAX2	rs11190709	rs7072737	chr10:102556175	G/A	0.989	Proxy	BMI	5.87E-05	25673413
	rs11190709	rs10883543	chr10:102552752	T/G	1	Proxy	Alzheimers disease	6.50E-05	19734902
LINC00477-SOX5	rs4246224	rs17287293	chr12:24770878	G/A	1	Proxy	**Heart rate**	**3.00E-20**	23583979
	rs4246224	rs11047543	chr12:24788339	A/G	1	Proxy	**Electrocardiography**	**3.00E-13**	20062060
	rs4246224	rs11047543	chr12:24788339	A/G	1	Proxy	**PR interval**	**3.00E-13**	20062060
	rs4246224	rs17287293	chr12:24770878	G/A	1	Proxy	**PR duration**	**4.91E-13**	23583979
	rs4246224	rs17287293	chr12:24770878	G/A	1	Proxy	**RR interval**	**5.70E-11**	20639392
	rs4246224	rs17287293	chr12:24770878	G/A	1	Proxy	Atrial fibrillation	2.38E-07	23583979
SYT10	rs1343676	rs1343676	chr12:33537387	C/T	1	Lead	**Heart rate**	**6.02E-11**	23583979
HMGA2	rs1480470	rs899844	chr12:66398933	A/C	0.983	Proxy	**Height**	**2.70E-14**	25282103
	rs1480470	rs4026608	chr12:66394664	C/T	0.91	Proxy	**Aortic root size**	**1.75E-09**	19584346
	rs1480470	rs899844	chr12:66398933	A/C	0.983	Proxy	Birthlength	1.67E-05	25281659
TCF4	rs1125313	rs1262465	chr18:52857732	A/G	0.909	Proxy	Neuroticism	1.66E-07	27089181
NDUFA11	rs12974991	rs12980262	chr19:5893058	A/G	1	Proxy	Autism	3.98E-05	20663923

Supplementary Table 9: HR response to exercise loci associations with other traits using PhenoScanner
The look-up results from the lead HR SNP or proxy SNPs in high LD (r2 ≥ 0.8) from the 1000 Genome Project are indicated. SNPs are ordered by chromosomal position, and only results with P value ≤ 5 x 10-8 are included. If there were multiple results for the same trait, the variant from the earliest publication is shown. Proxy variants with additional traits that were not associated with the lead variants are also included. If multiple proxy SNPs were available, the proxy SNP with the highest LD was chosen. EA: Effect allele; r2: A measure of the linkage disequilibrium between

the proxy and lead SNP; Type: Whether the variant is the lead or proxy variant; P: P-value for the association between the variant and the trait; PMID: PubMed ID. * indicates locus identified by sex-stratified analyses. Bold type indicates association with an ECG trait. The locus name indicates the gene that is in the closest proximity to the most associated SNP.

**Locus**	**Trait**	**SNP**	**CHR**	**BP**	**Candidate genes within 5kb**	**eQTL**	**Missense variant**	**Hi-C interactor genes**	**Candidate gene(s) at locus**	**Mouse model and support**
RNF220	Both	rs272564	1	45012273	MIR5584; RNF220			RNF220; ERMAP; ERI3; KLF17; DMAP1	RNF220	
BCL11A	Recovery	rs1372876	2	60025963	LINC01122			BCL11A; VRK2; MIR4432; LINC01122	BCL11A	http://www.informatics.jax.org/marker/key/26923
BCL11A†	Recovery	rs2539671	2	59879267	LINC01122			BCL11A; VRK2; MIR4432; LINC01122	BCL11A	http://www.informatics.jax.org/marker/key/26923
CCDC141	Exercise	rs10497529	2	179839888	CCDC141		CCDC141		CCDC141	
SCN10A	Both	rs7433723	3	38784957	SCN10A		SCN10A	SCN5A; SCN11A; OXSR1; XYLB; OG; ACVR2B;	SCN10A, SCN11A	http://www.informatics.jax.org/m

								CTNNB1; MOBP; WDR48; STAC; EXOG		arker/key/13354
CNTN3	Recovery	rs6549649	3	74786491	CNTN3	CNTN3		CNTN3; PDZRN3; ROBO2	CNTN3	
SNCA1P	Both	rs4836027	5	121866990	MGC32805			PRDM6; SNCAIP; CEP120; PPIC; SRFBP1; CSNK1G3; SNX24; ZNF608; LOC100505841	PRDM6, SNCAIP	
PPIL1	Exercise	rs236352	6	36817113	PPIL1			CPNE5; MIR3925; PIM1; STK38; TMEM217; CMTR1; TCP11; BTBD9; TBC1D22B; PNPLA1; GLO1; GLP1R	BTBD9	http://www.informatics.jax.org/marker/key/53355
ACHE	Recovery	rs3757868	7	100482720	ACHE, SSRT, UFSP1, SLC12A9	ACHE, SSRT	UFSP1	LOC100289561	ACHE, SSRT	http://www.informatics.jax.org/marker/key/36

CAV2	Both	rs28495552	7	116113744	CAV1; CAV2			CAV1; TES; CAPZA2; MET; MDFIC; FOXP2; TFEC; ST7; ST7-AS2; MIR3666; ASZ1; ST7-OT4	CAV2, CAV1	
CHRM2	Recovery	rs6943656	7	136639436	CHRM2; LOC349160			MIR490; CHRM2; SLC13A4; STMP1; PTN; C7orf55-LUC7L2	CHRM2	http://www.informatics.jax.org/marker/key/982
RP1L1	Exercise	rs58065122	8	10526186	RP1L1, C8orf74			N.A.	RP1L1	
PAX2	Both	rs11190709	10	102552663	PAX2			PAX2; SEC31B; SLF2	PAX2, SEC31B	
LINC00477 - SOX5	Both	rs4246224	12	24784139	LINC00477			PAX2; SEC31B; SCD; SLF2; SH3PXD2A	SOX5, LINC00477	
SYT10	Both	rs1343676	12	33537387	SYT10			SYT10; TSPAN11; CAPRIN2	SYT10	http://www.informatics.jax.org/marker/key/46756
ALG10B	Recovery	rs4533105	12	38214611	ALG10B			SLC2A13; ALGB10; CNTN1; KIF21A; CPNE8; ABCD2;	ALG10B; CNTN1	http://www.informatics.jax.org/marker/k

										ey/73599
HMGA2	Exercise	rs1480470	12	66412130	HMGA2			FLJ41278; WIF1; LEMD3; TBC1D30; MSRB3; CPM; AVPR1A; XPOT; TMEM5; HMGA2	LEMD3, FLJ41278	
MED13L	Recovery	rs11067773	12	116228495	MED13L			TBX3; HRK; TBX5; FBXW8	TBX3	http://www.informatics.jax.org/marker/key/13764
RGS6	Recovery	rs150330648	14	72844765	RGS6			RGS6	RGS6	http://www.informatics.jax.org/marker/MGI:1354730
RGS6‡	Recovery	rs17180489	14	72885471	RGS6			RGS6; DPF3; PCNX1	RGS6	
MCTP2	Both	rs12906962	15	95312071	LOC440311			N.A.	LOC440311, FAM174B	http://www.informatics.jax.org/marker/MGI:1

										354730
TCF4	Exercise	rs1125313	18	52859261	TCF4			WDR7; TCF4; LINC01539; LINC-ROR; NEDD4L; STARD6	WDR7, TCF4	http://www.informatics.jax.org/marker/key/13790
POP4	Exercise	rs7255293	19	30104198	POP4			N.A.	POP4	
NDUFA11	Recovery	rs12974991	19	5894584	FUT5		NDUFA11	N.A.	CAPDS	http://www.informatics.jax.org/marker/key/44726

Supplementary Table 10: Candidate genes for Goprofiler analysis

Abbreviations: SNP: single-nucleotide polymorphism, CHR: chromosome, BP: Base pair position, based on HG built 18, eQTL: evidence of an expression quantitative trait locus; Hi-C interactor genes are candidate genes indicated from Supplementary Table 15; N.A. indicates no interacting genes. † and ‡ indicates secondary signals. Column K provides URL links to mouse models with cardiovascular and neural phenotypes.

	HR values Validated Group			HR values Non-Validated Group		
	**Mean**	**SD**	**n**	**Mean**	**SD**	**n**
**Rest**	71.92	11.78	52397	71.38	11.71	14447
**Peak exercise**	114.65	14.06	52365	114.21	14.52	14435
**Recovery**	83.14	14.20	52250	82.29	14.17	14415

Supplementary Table 11: Descriptives of resting HR, peak HR and recovery HR for the validated group (subjects with ECG data) and the non-validated group (subjects without ECG data).
n = number, SD: Standard Deviation.
