# Supplementary material for: Thirty loci identified for heart rate response to exercise and recovery implicate autonomic nervous system
Source: Nat Commun. 2018 May 16;9:1947. doi: 10.1038/s41467-018-04148-1 (PMC5955978; doi:10.1038/s41467-018-04148-1)
Supplement: Supplementary file 2 — Description of Additional Supplementary Files [file 41467_2018_4148_MOESM2_ESM.pdf]

Description of Additional Supplementary Files

Supplementary Data 1: Genome-wide significant SNPs for HR response to recovery in the discovery sample

Abbreviations: SNP: single-nucleotide polymorphism, Chr: Pos: Chromosome: Position, based on HG build 18, EA: Effect allele, AA: Alternate allele, EAF: Effect allele frequency, INFO: imputation information score, β: Beta in beats per minute, se: Standard Error, N: number of participants, *P*: P-value.

Supplementary Data 2: Association results of known resting HR and HRV loci in the HR response to exercise full dataset GWAS

Abbreviations: SNP: single-nucleotide polymorphism, CHR: chromosome, BP: Base pair position, based on HG built 18, EA: effect allele, AA: alternate allele, EAF: effect allele frequency, β: Beta in beats per minute, se: Standard Error, N: effective number of participants, P: P-value, HRV: Heart rate variability. NA - no proxies available in dataset. The locus name indicates the gene that is in the closest proximity to the most associated SNP.

Bold rows indicate genome-wide significant in the HR response to exercise full dataset GWAS.

Supplementary Data 3: Association results of known resting HR and HRV loci for HR response to recovery in the full dataset GWAS

Abbreviations: SNP: single-nucleotide polymorphism, CHR: chromosome, BP: Base pair position, based on HG built 18, EA: effect allele, AA: alternate allele, EAF: effect allele frequency, β: Beta in beats per minute, SE: Standard Error, N: effective number of participants, P: P-value, HRV: Heart rate variability. NA - no proxies available in dataset. The locus name indicates the gene that is in the closest proximity to the most associated SNP.

Bold rows indicate genome-wide significant in the HR response to exercise full dataset GWAS.

Supplementary Data 4: Association results of known resting HR and HRV loci for resting HR in the full dataset GWAS

Abbreviations: SNP: single-nucleotide polymorphism, CHR: chromosome, BP: Base pair position, based on HG built 18, EA: effect allele, AA: alternate allele, EAF: effect allele frequency, β: Beta in beats per minute, SE: Standard Error, N: effective number of participants, P: P-value, HRV: Heart rate variability. NA - no proxies available in dataset. PMID indicates PubMed ID. The locus name indicates the gene that is in the closest proximity to the most associated SNP.

Bold rows indicate genome-wide significant in the HR response to exercise full dataset GWAS.

Supplementary Data 5: Expression quantitative trait locus (eQTL) analysis for HR response to exercise and to recovery traits

HR response to exercise and to recovery variants with significant eQTLs and their corresponding genes are indicated. The results from proxy variants, with high LD (r2 ≥ 0.8) with the lead variant in the 1000 Genome Project were included if there was tissue expression data in addition to the lead variant. Results were filtered to those reaching a P value ≤ 5 x 10-8. The source was Genotype-Tissue Expression (GTEx) Consortium, PubMed ID is 25954001. r^2: A measure for the linkage disequilibrium between the proxy and lead SNPs; *P*: P value for the association between the variant and RNA tissue expression.

Supplementary Data 6: Long-range interactors in heart, adrenal, brain tissue and neural progenitor cells

Results are presented for all SNPs in LD $r^2 \geq 0.8$ with lead SNPs found in this study that have a functional score ≤ 5, and the locus has at least one significant Hi-C interaction.
